# Supplementary material for: ERFVII action and modulation through oxygen-sensing in Arabidopsis thaliana
Source: Nat Commun. 2023 Aug 3;14:4665. doi: 10.1038/s41467-023-40366-y (PMC10400637; doi:10.1038/s41467-023-40366-y)
Supplement: Supplementary file 1 — Supplementary Information [file 41467_2023_40366_MOESM1_ESM.pdf]

**Supplementary Figure 1**

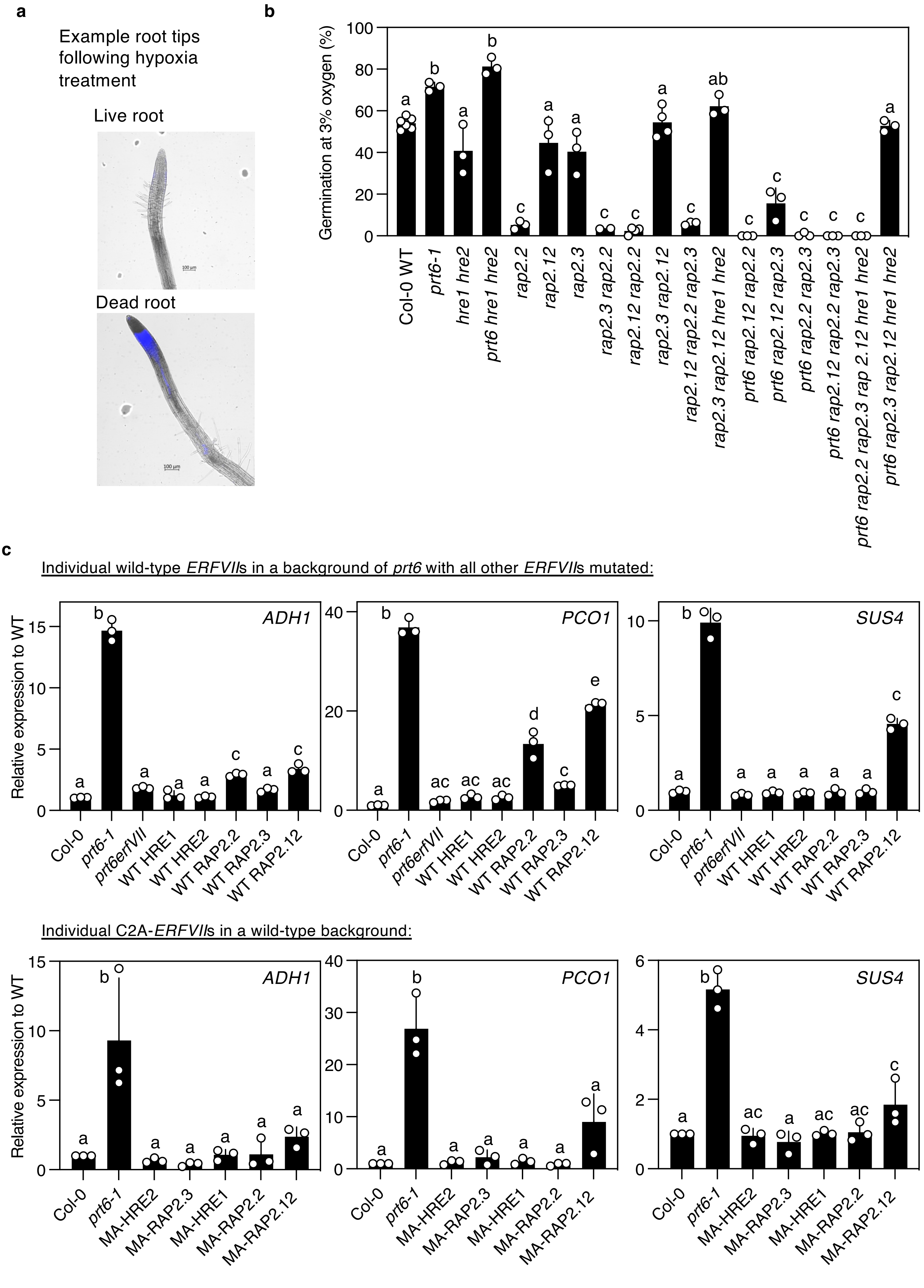

**Supplementary Figure 1: Influence of *ERFVII*s on hypoxia tolerance**

S1a: Trypan blue staining to show death of root tip cells comparing live (no staining) to dead (blue staining). Merged image with bright field is shown fortwo root tips following hypoxia treatment.

S1b: Germination under 3% ambient oxygen for wild-type and mutant seeds.

S1c: Expression of hypoxia-related genes *ADH1*, *PCO1* and *SUS4* in WT and mutant or transgenic seedlings in normoxia.

Means are plotted with SD, data were analysed by one-way ANOVA and different letters indicate significant differences (p-value < 0.05) (n= 3).

**Supplementary Figure 2**

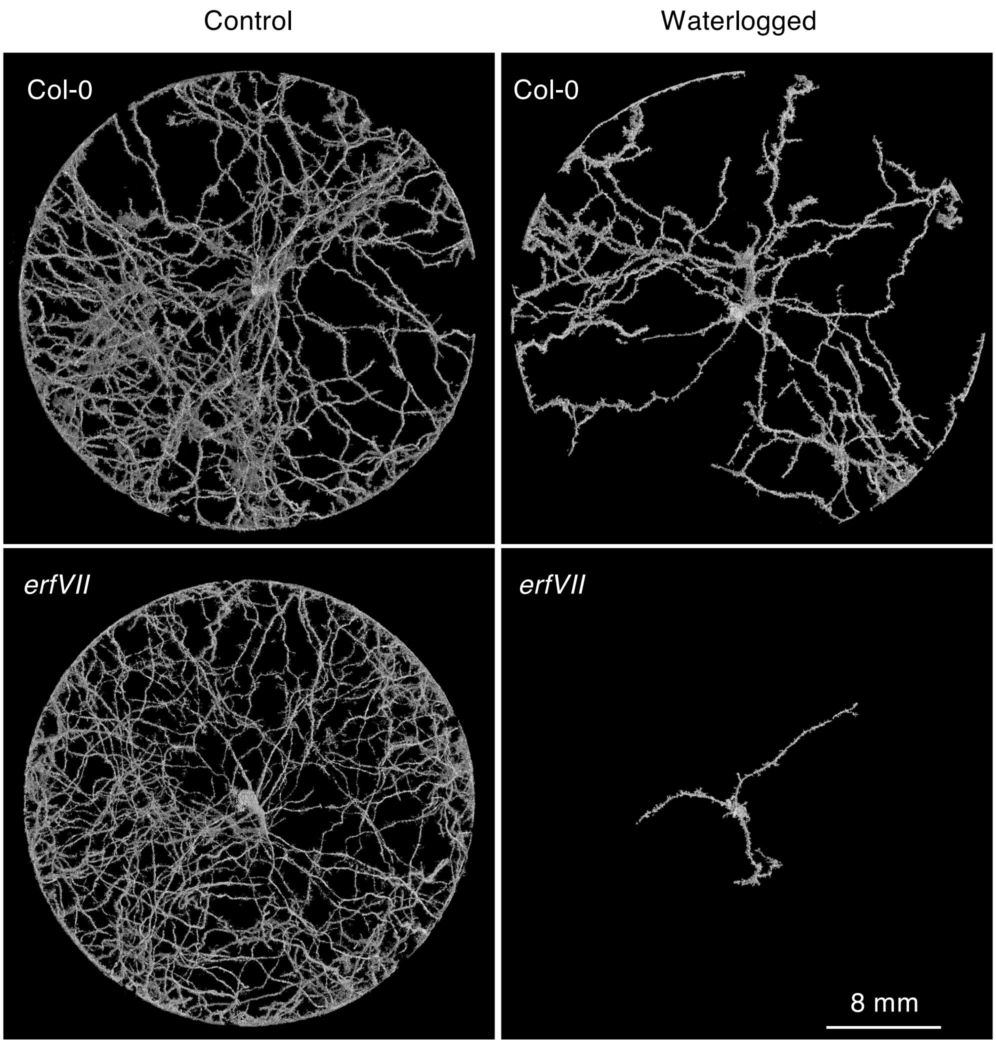


**Supplementary Figure 2: *ERFVII* role on root structure in waterlogged soil**

Representative 3D rendered X-ray computed micro-tomography top view images of wild type (Col-0) and *erfVII* mutant roots grown in sandy-clay loam soil in control conditions or following waterlogging treatment for 7 days. Scale bar is 8 mm.

## Supplementary Figure 3

a

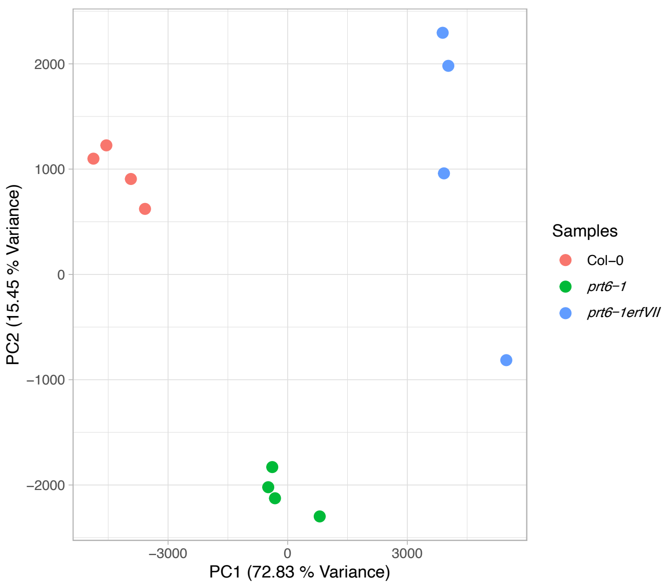


b

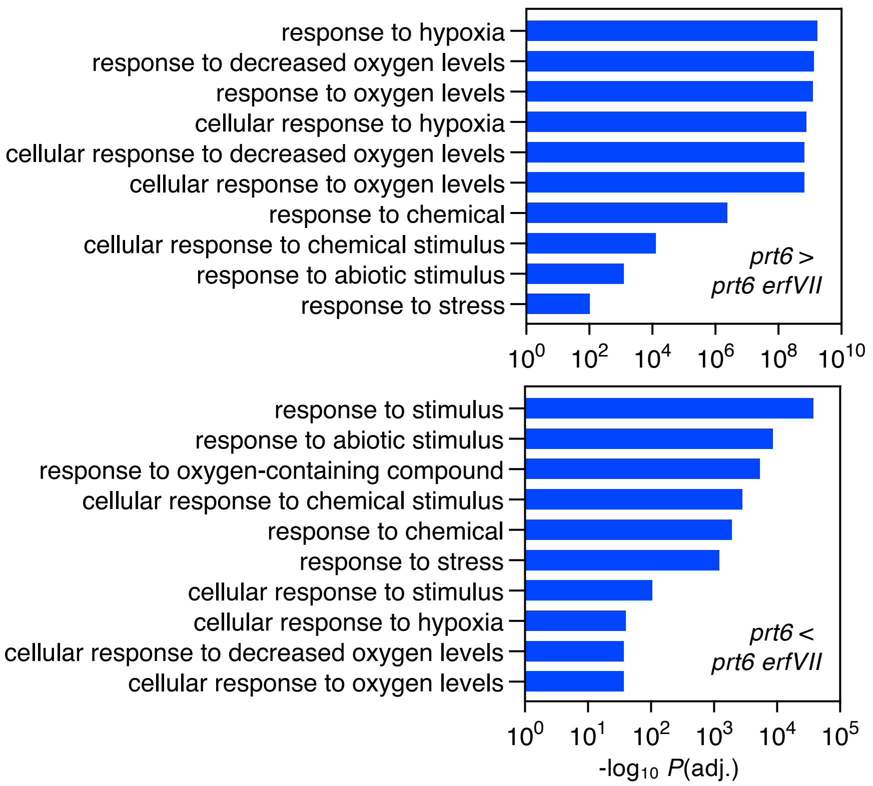


**Supplementary Figure 3: Transcriptome analysis of ERFVII effect on genome expression**
a. Principal component analysis for replicates of RNA samples used in transcriptome comparisons. Principal components 1 and 2 are shown, that represent 88.28% of total variance. b. Overlap of genes from *prt6* vs *prt6 erfVII* that both contain HRPE in promoter or genomic coding regions and are bound by HRE2 in ChIP experiments with seedlings (Lee and Bailey-Serres Plant Cell 31, 2573-2595, doi:10.1105/tpc.19.00463 (2019). $p<0.05$, Fisher's one-tailed test. See Table S11.

**Supplementary Figure 4**

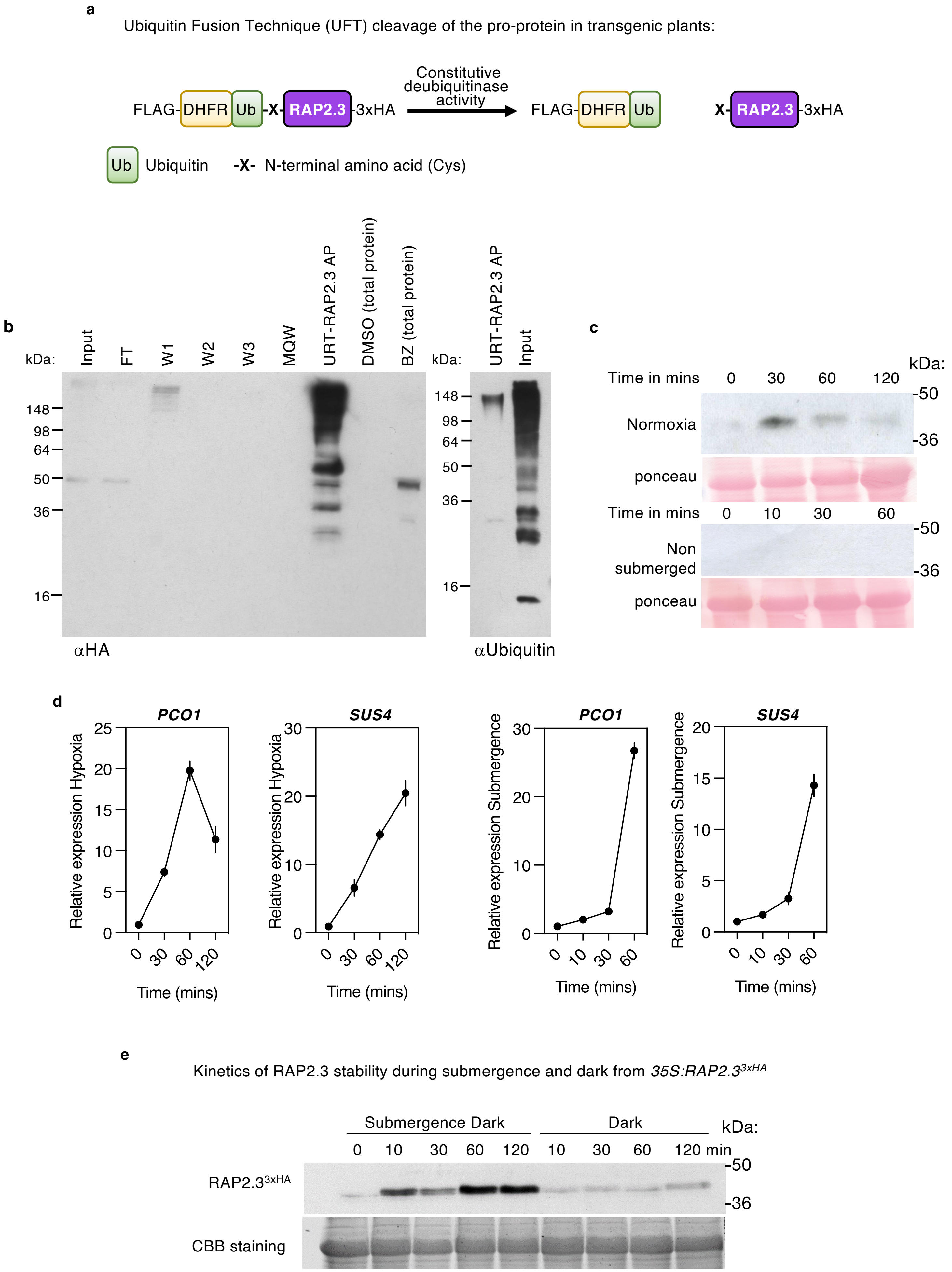

**Supplementary Figure 4: Gene expression and protein abundance in response to reduced oxygen conditions**

a. Diagram of the UFT:RAP2.3$^{3xH}$A construct used to analyse stability of RAP2.3. Ub, ubiquitin; X-, first amino-acid of the test protein.

b. Western blots showing polyubiquitination of RAP2.3$^{3xHA}$ in vivo. Transgenic plants expressing RAP2.3$^{3XHA}$ were used for immunoprecipitation with anti-HA magnetic beads. Anti-HA and anti-ubiquitin antibodies were used to detect polyubiquitinated RAP2.3 in vivo.  Input, total protein extract; FT, flow through; W1-W3, phosphate buffer saline washes; MWQ, molecular grade water wash.

c. Western blot analysis of RAP2.3$^{3xH}$A abundance in control WT seedlings for hypoxia (normoxia in Fig. 4c) and non-submerged (submerged in Fig. 4c) seedlings. Ponceau staining is shown.

d. Time-course of expression of low-oxygen responsive transcripts *PCO1* and *SUS4* in WT seedlings in response to submergence or hypoxia treatment. Means are plotted with SD (n=3).

e. Time-course Western blot analysis of RAP2.3$^{3xHA}$ abundance in WT seedlings in response to submergence treatment, derived from the *35S:RAP2.3*$^{3xHA}$ transgene 15. CBB Coomassie brilliant blue stained gel.

## Supplementary Figure 5

**a**

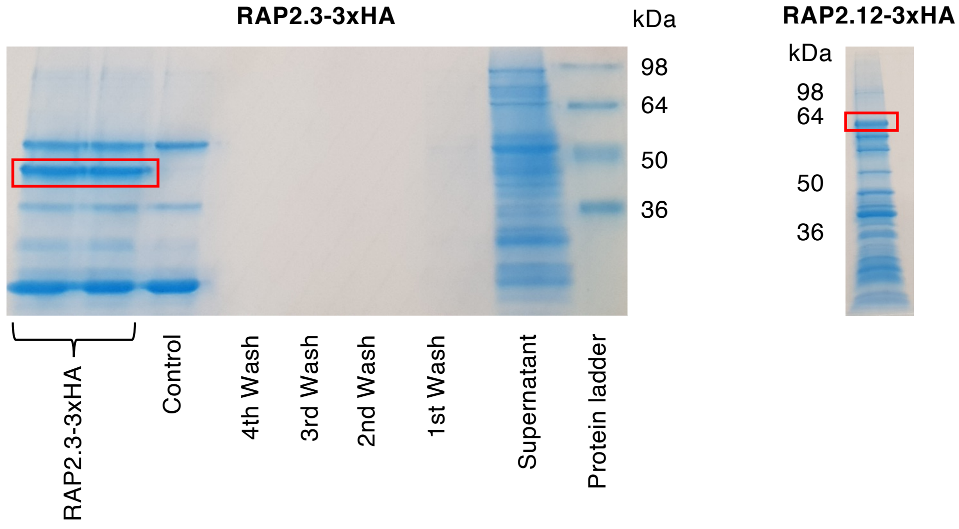


**b**

**RAP2.3**

**RCGGAIISDY APLVTK**AKGR **KLTAEELWSE**
**LDASAADDFW G**FYSTSKLHP TNQVNVKEEA
VKKEQATEPG KRRKRKNVYR GIRKRPWGKW
AAEIRDPRKG VR**VWLGTFNT AEEAAMAYDV**
**AAK**QIRGDKA KLNFPDLHHP PPPNYTPPPS
SPRSTDQPPA KKVCVVSQSE SELSQPSFPV
ECIGFGNGDE FQNLSYGFEP DYDLKQQISS
LESFLELDGN TAEQPSQLDE SVSEVDMWML
DDVIASYE

**RAP2.12**

**RCGGAIISDF IPPPRSRRVT SEFIWPDLKK** NLKGSKKSSK
NR**SNFFDFDA EFEADFQGFK** DDSSIDCDDD FDVGDVFADV
KPFVFTSTPK **PAVSAAAEGS VFGKKVTGLD GDAEK**SANRK
RKNQYRGIRQ RPWGKWAAEI RDPREGAR**IW LGTFK**TAEEA
ARAYDAAARR IRGSKAKVNF PEENMKANSQ KRSVKANLQK
PVAKPNPNPS PALVQNSNIS FENMCFMEEK HQVSNNNNNQ
FGMTNSVDAG CNGYQYFSSD QGSNSFDCSE FGWSDQAPIT
PDISSAVINN NNSALFFEEA NPAKKLKSMD FETPYNNTEW
DASLDFLNED AVTTQDNGAN PMDLWSIDEI HSMIGGVF

**c**

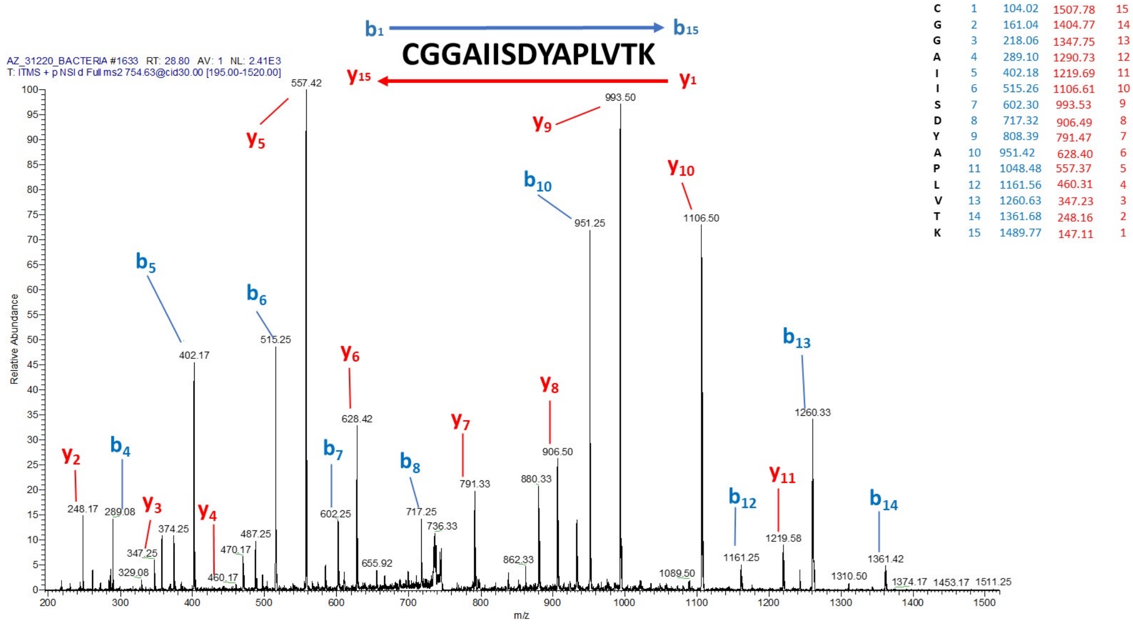


| | | b | y | |
|---|---|---|---|---|
| C | 1 | 104.02 | 1507.78 | 15 |
| G | 2 | 161.04 | 1404.77 | 14 |
| G | 3 | 218.06 | 1347.75 | 13 |
| A | 4 | 289.10 | 1290.73 | 12 |
| I | 5 | 402.18 | 1219.69 | 11 |
| I | 6 | 515.26 | 1106.61 | 10 |
| S | 7 | 602.30 | 993.53 | 9 |
| D | 8 | 717.32 | 906.49 | 8 |
| Y | 9 | 808.39 | 791.47 | 7 |
| A | 10 | 951.42 | 628.40 | 6 |
| P | 11 | 1048.48 | 557.37 | 5 |
| L | 12 | 1161.56 | 460.31 | 4 |
| V | 13 | 1260.63 | 347.23 | 3 |
| T | 14 | 1361.68 | 248.16 | 2 |
| K | 15 | 1489.77 | 147.11 | 1 |

**Supplementary Figure 5 (cont)**

**d**

**RAP2.3: RC$^{3ox}$GGAIISDYAPLVTK**

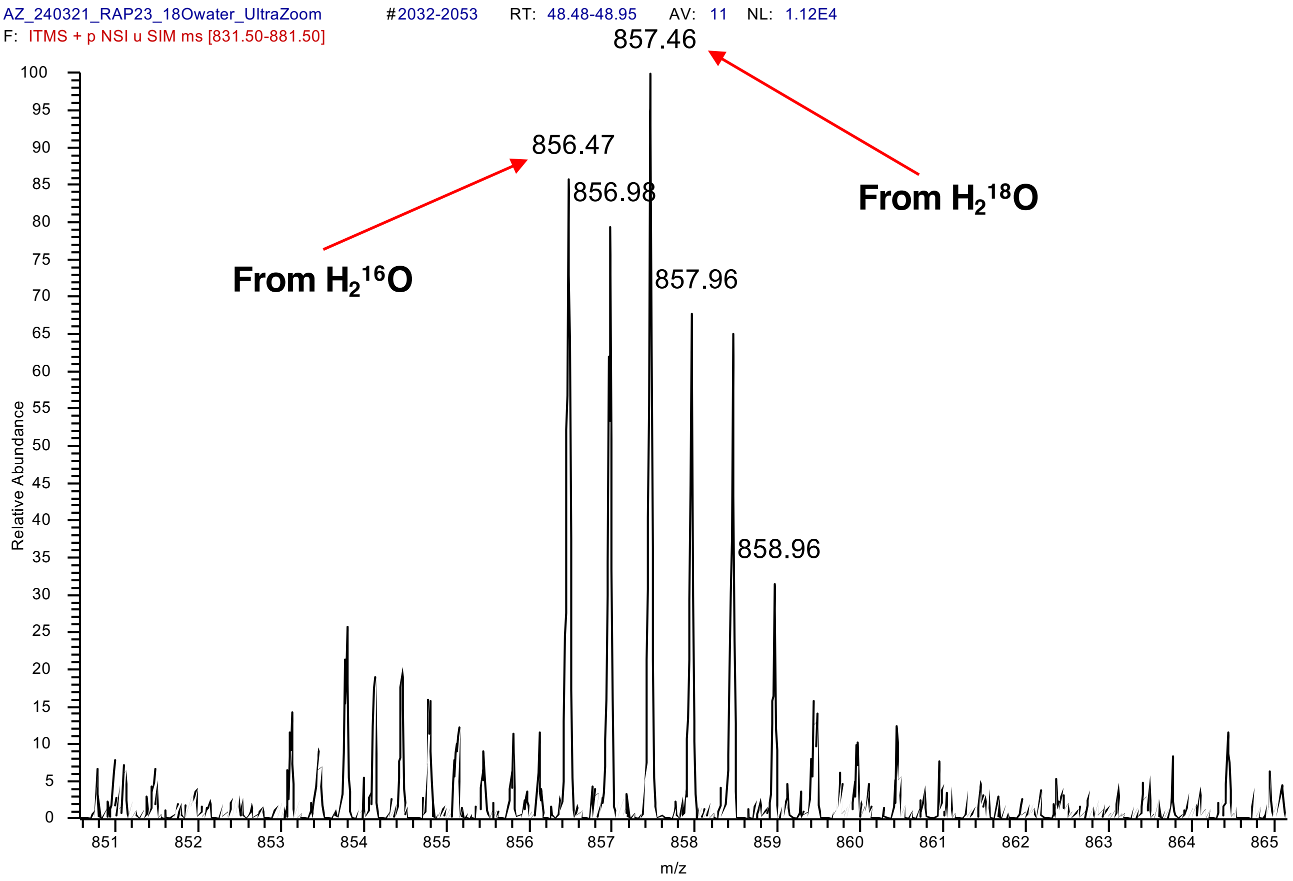


**e**

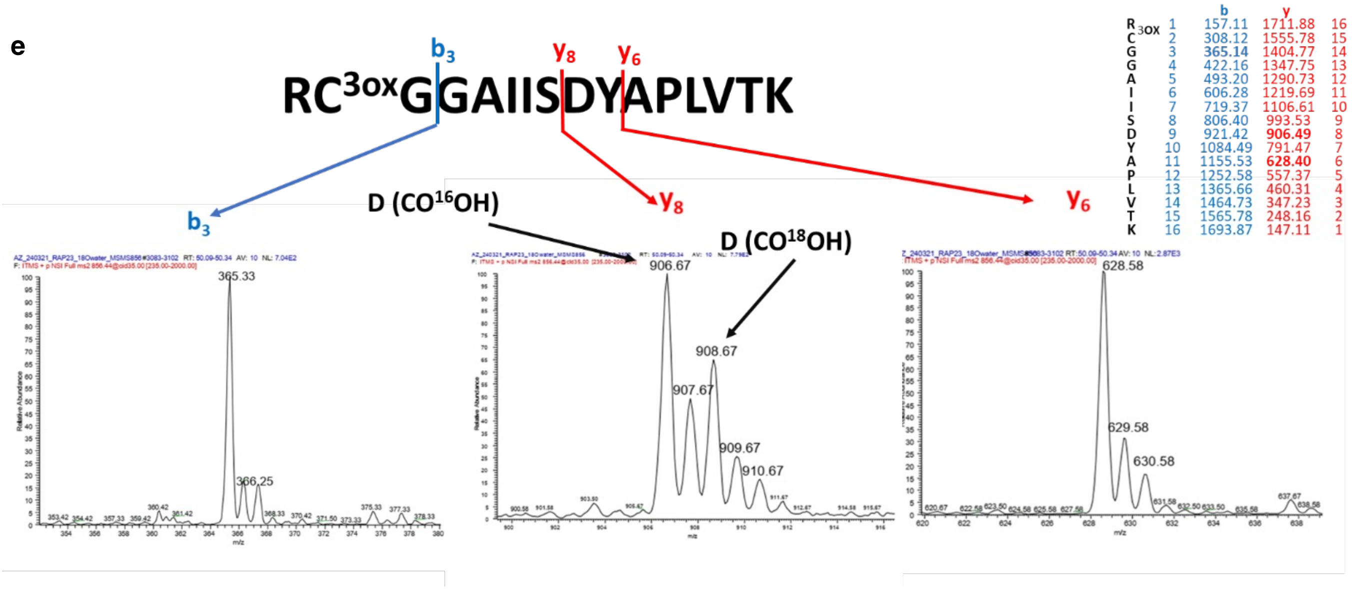


**Supplementary Figure 5: Analysis of the chemical structure at the amino-terminus of RAP2.3 and RAP2.12**

a. Coomassie Blue stained SDS-PAGE gel showing RAP2.3$^{3xHA}$ and RAP2.12$^{3xHA}$ purified following coupled in vitro transcription/translation in a wheat germ extract. The purification scheme including 4 washed is shown for RAP3.2$^{3xHA}$, for RAP2.12$^{3xHA}$ just the final purified eluate is shown. Bands excised for MS analysis are indicated in red boxes.

b. Identification of HA-affinity purified protein bands as RAP2.3 and RAP2. 12 based on SearchGUI database searches of LC-MS/MS data. High quality MS/MS hits for 9 peptides were recorded for each protein, and the coverage indicated in red on each sequence. Associated spectra in Supplementary Data 1.

c. MS/MS spectra of the Nt tryptic peptides of RAP2.3HIS ([M+2H]2+ = *m/z* 754.4) following expression in *E. coli.*

d. LC-MS analysis of the tryptic digest of RAP2.3$^{3xHA}$ synthesised in the presence of 40% H218O.

e. RAP2.3$^{3xHA}$ MS/MS analysis on the [M+2H]2+ precursor ions at *m/z* 856.5/857.5 showing $^{18}O$ incorporation into the carboxylate group of Asp-9 and no $^{18}O$ incorporation into Cys2.
