# Supplementary material for: ERFVII action and modulation through oxygen-sensing in Arabidopsis thaliana
Source: Nat Commun. 2023 Aug 3;14:4665. doi: 10.1038/s41467-023-40366-y (PMC10400637; doi:10.1038/s41467-023-40366-y)
Supplement: Supplementary file 3 — Description of Additional Supplementary Files [file 41467_2023_40366_MOESM3_ESM.pdf]

Description of Additional Supplementary Files

Supplementary Data 1. Statistically significant differentially regulated genes between Col-0 and *prt6 erfVII*

Supplementary Data 2. Statistically significant differentially regulated genes between Col-0 and *prt6-1*

Supplementary Data 3. Statistically significant differentially regulated genes between *prt6-1* and *prt6 erfVII*

Supplementary Data 4. GO and KEGG pathway analysis for *prt6* > *prt6 erfVII* genes more than 2 fold upregulated; Fisher's one-tailed test ($p<0.05$).

Supplementary Data 5. GO and KEGG pathway analysis for *prt6* < *prt6 erfVII* genes more than 2 fold upregulated; Fisher's one-tailed test ($p<0.05$).

Supplementary Data 6. Fold difference transcript levels in transcriptome comparisons for a set of 49 core hypoxia genes.

Supplementary Data 7. Identification of HRPE elements in the genomic locus region of genes *prt6* > *prt6 erfVII*.

Supplementary Data 8. Identification of HRPE elements in the genomic locus region of genes *prt6* < *prt6 erfVII*.

Supplementary Data 9. Identification of HRPE elements in the -1000 promoter region of genes *prt6* > *prt6 erfVII*.

Supplementary Data 10. Identification of HRPE elements in the -1000 promoter region of *genes prt6* < *prt6 erfVII*.

Supplementary Data 11. Overlap of genes from *prt6 vs prt6 erfVII* that both contain HRPE in promoter or genomic coding region and are bound by HRE2 in ChIP; Fisher's one-tailed test ($p<0.05$).

Supplementary Data 12. FASTA sequences and RAP2.3 and RAP2.12 search spectra.

Supplementary Data 13. List of oligonucleotide primers used for qRT-PCR.

Supplementary Movie 1. 3D rendered X-ray computed micro-tomography movie showing a comparison between four-week-old wild-type (Col-0) and *erfVII* mutant roots grown in sandyclay loam soil under control conditions.

Supplementary Movie 2. 3D rendered X-ray computed micro-tomography movie showing a comparison between four-week-old wild-type (Col-0) and *erfVII* mutant roots grown in sandyclay loam and waterlogged for 7 days.
