# Supplementary material for: ERFVII action and modulation through oxygen-sensing in Arabidopsis thaliana
Source: Nat Commun. 2023 Aug 3;14:4665. doi: 10.1038/s41467-023-40366-y (PMC10400637; doi:10.1038/s41467-023-40366-y)

**Supplementary Data 12:** **FASTA sequences and** **RAP2.3 and RAP2.12 search spectra**

A. FASTA sequences for RAP2.3 and RAP2.12:

>RAP2.3

MCGGAIISDY APLVTKAKGR KLTAEELWSE LDASAADDFW GFYSTSKLHP TNQVNVKEEA VKKEQATEPG KRRKRKNVYR GIRKRPWGKW AAEIRDPRKG VRVWLGTFNT AEEAAMAYDV AAKQIRGDKA KLNFPDLHHP PPPNYTPPPS SPRSTDQPPA KKVCVVSQSE SELSQPSFPV ECIGFGNGDE FQNLSYGFEP DYDLKQQISS LESFLELDGN TAEQPSQLDE SVSEVDMWML DDVIASYE

>RAP2.12

RCGGAIISDF IPPPRSRRVT SEFIWPDLKK NLKGSKKSSK NRSNFFDFDA EFEADFQGFK DDSSIDCDDD FDVGDVFADV KPFVFTSTPK PAVSAAAEGS VFGKKVTGLD GDAEKSANRK RKNQYRGIRQ RPWGKWAAEI RDPREGARIW LGTFKTAEEA ARAYDAAARR IRGSKAKVNF PEENMKANSQ KRSVKANLQK PVAKPNPNPS PALVQNSNIS FENMCFMEEK HQVSNNNNNQ FGMTNSVDAG CNGYQYFSSD QGSNSFDCSE FGWSDQAPIT PDISSAVINN NNSALFFEEA NPAKKLKSMD FETPYNNTEW DASLDFLNED AVTTQDNGAN PMDLWSIDEI HSMIGGVF

**B. RAP2.3 and RAP2.12 search spectra**

RAP2.3 expressed in wheat germ extract

**RCGGAIISDY APLVTK**AKGR **KLTAEELWSE LDASAADDFW G**FYSTSKLHP TNQVNVKEEA VKKEQATEPG KRRKRKNVYR GIRKRPWGKW AAEIRDPRKG VR**VWLGTFNT AEEAAMAYDV AAK**QIRGDKA KLNFPDLHHP PPPNYTPPPS SPRSTDQPPA KKVCVVSQSE SELSQPSFPV ECIGFGNGDE FQNLSYGFEP DYDLKQQISS LESFLELDGN TAEQPSQLDE SVSEVDMWML DDVIASYE


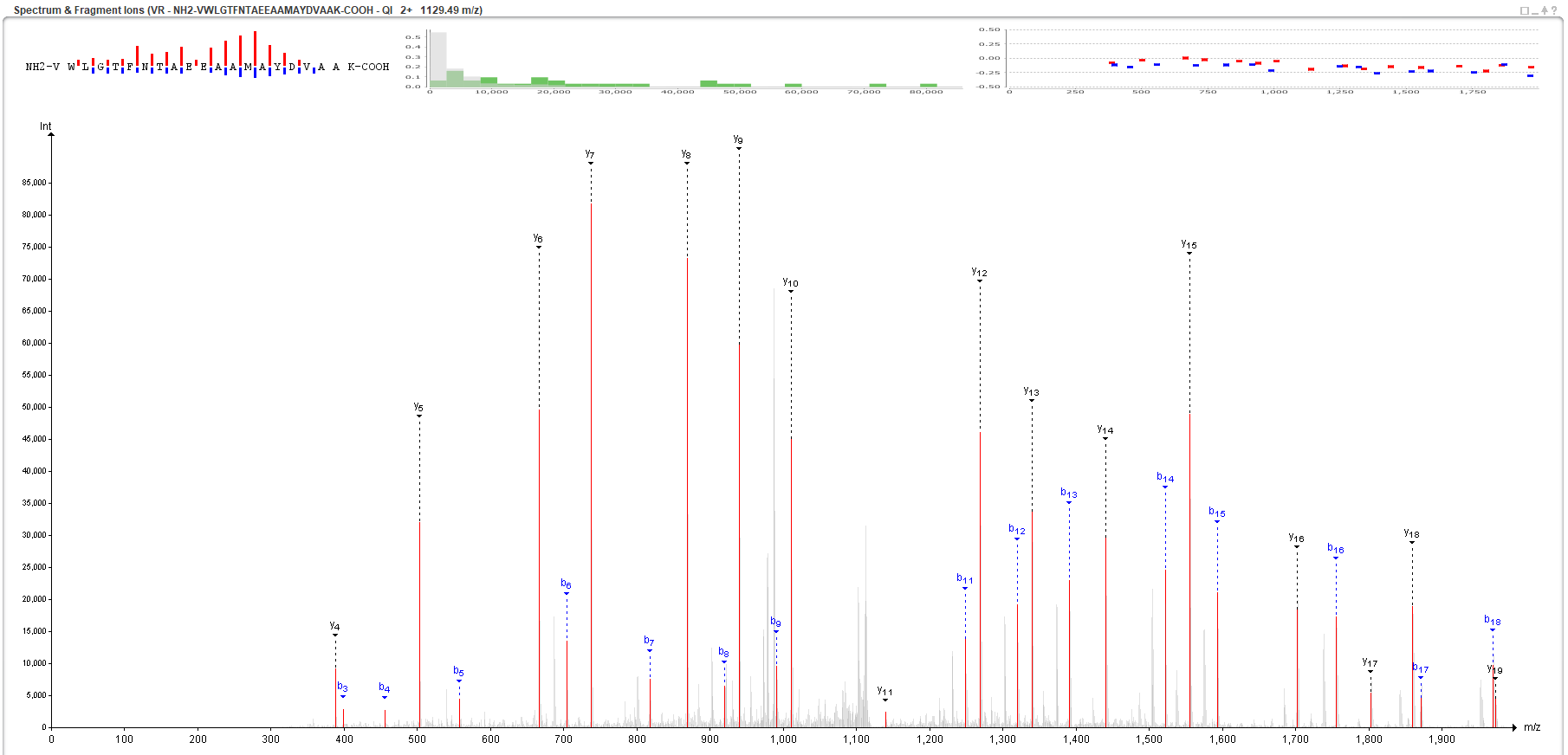


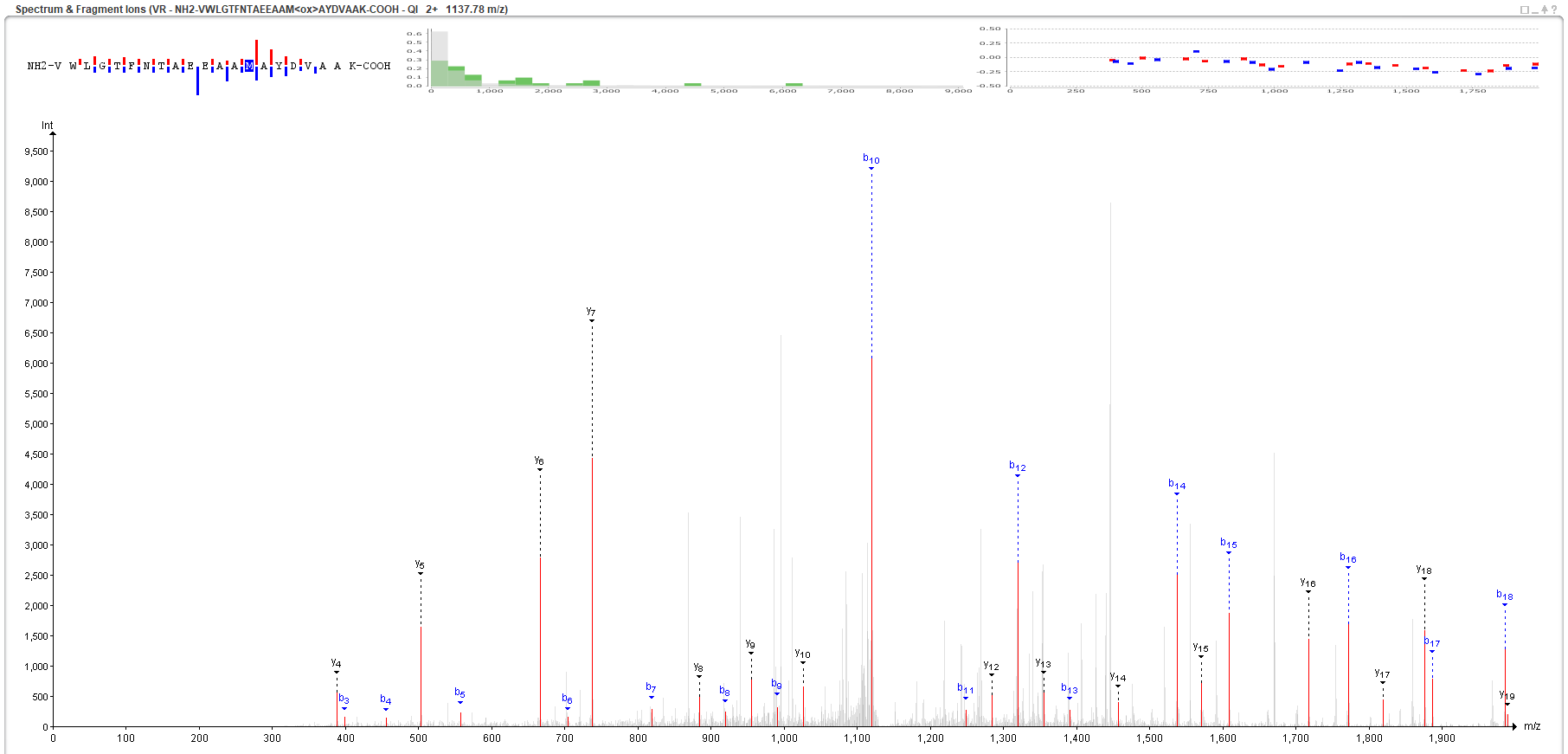


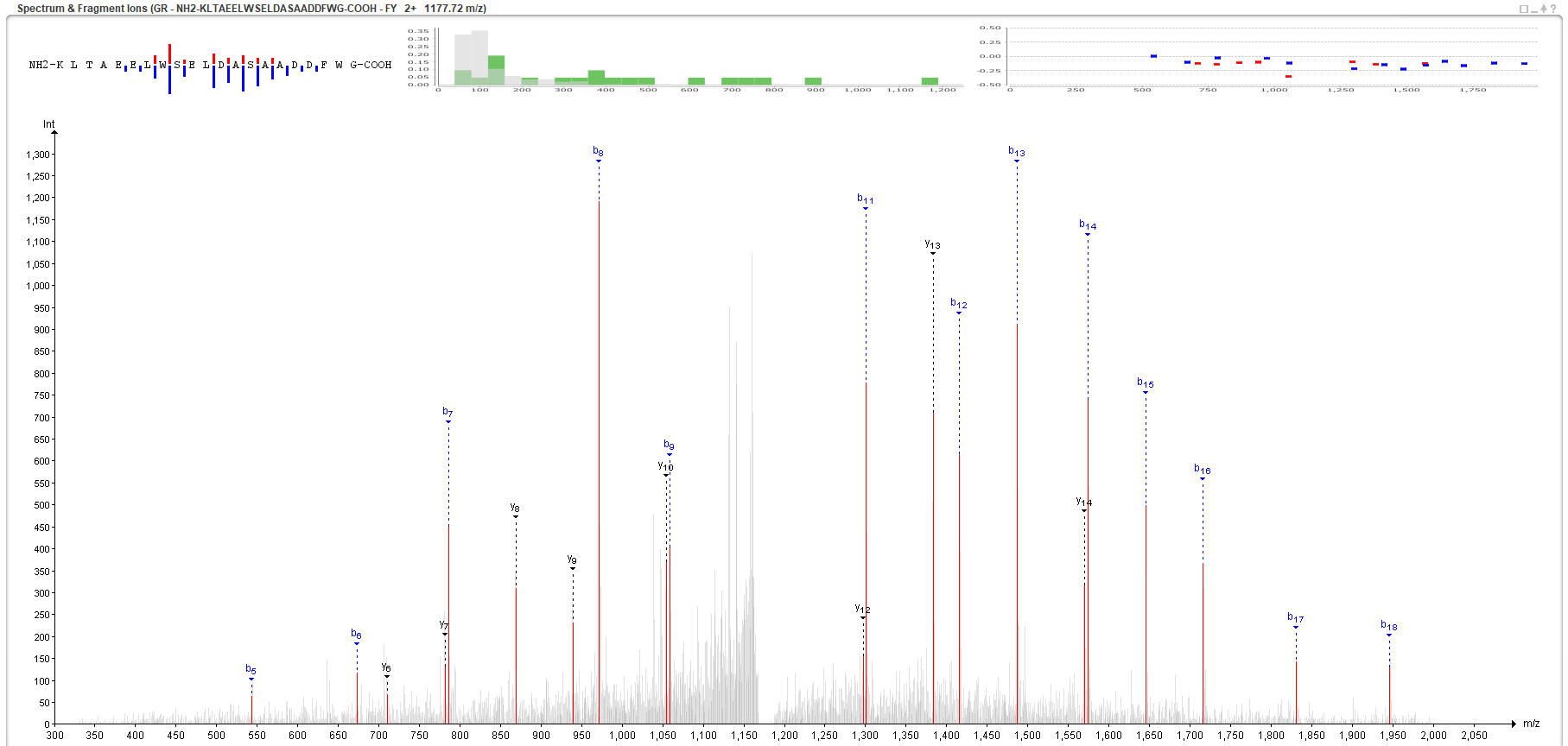


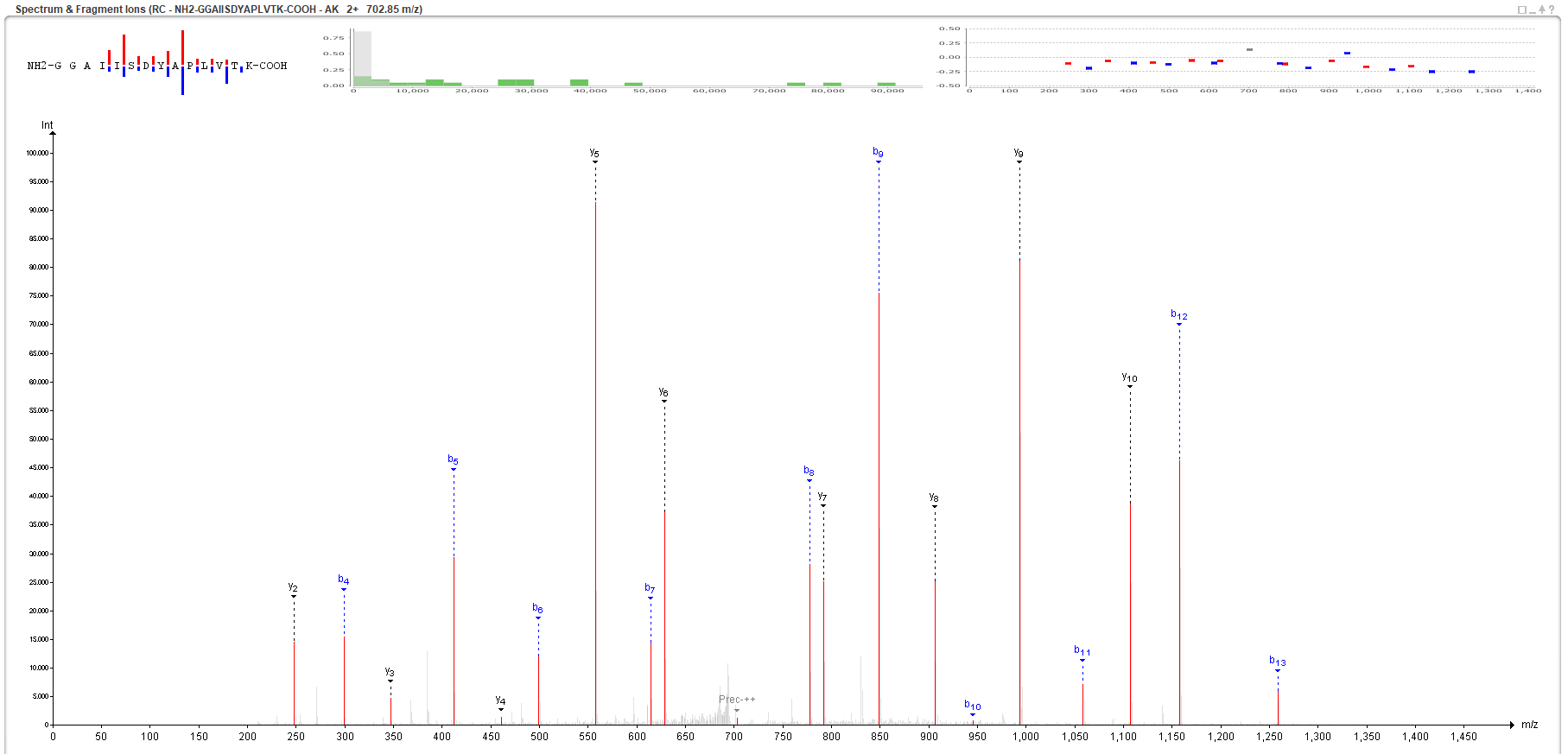


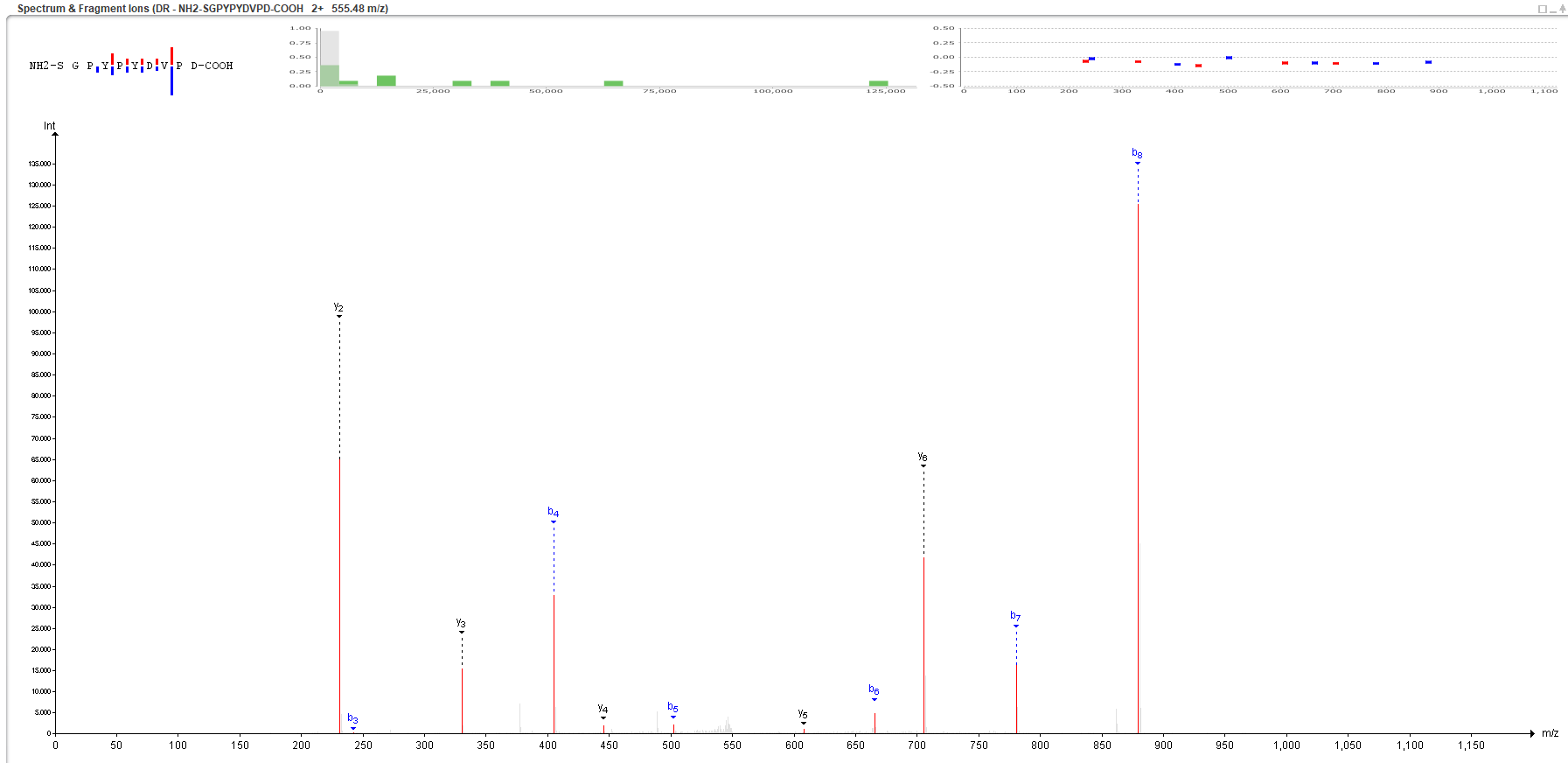


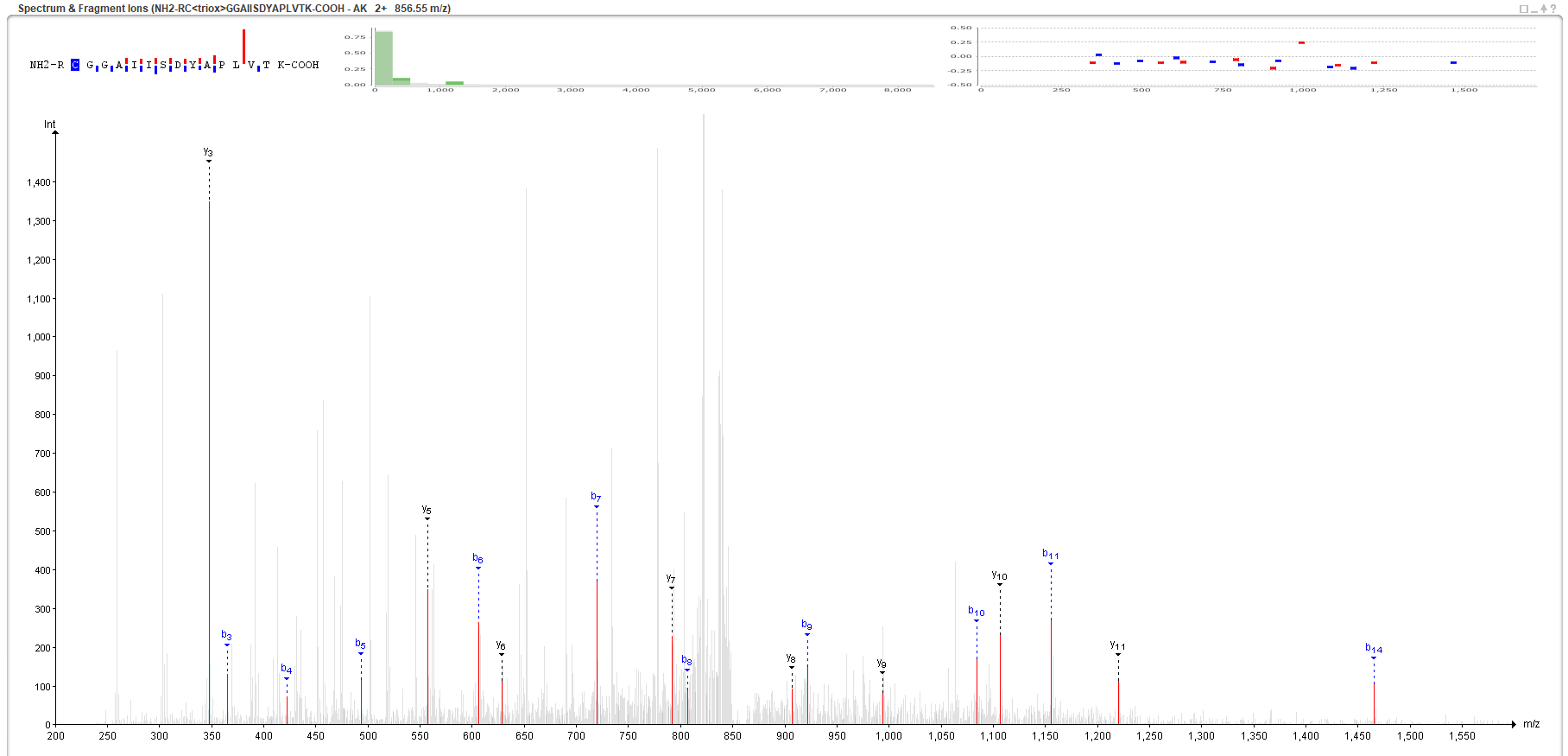


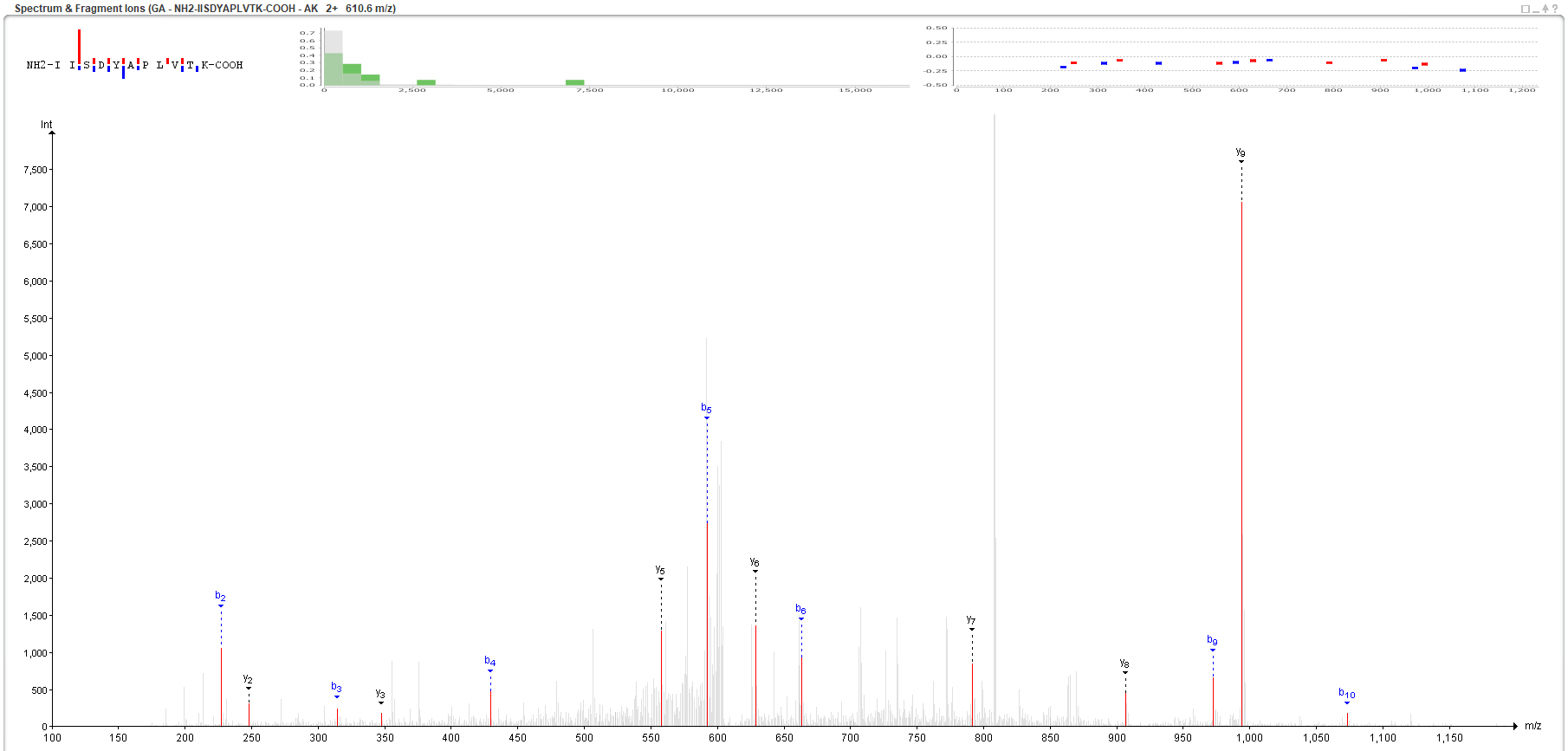


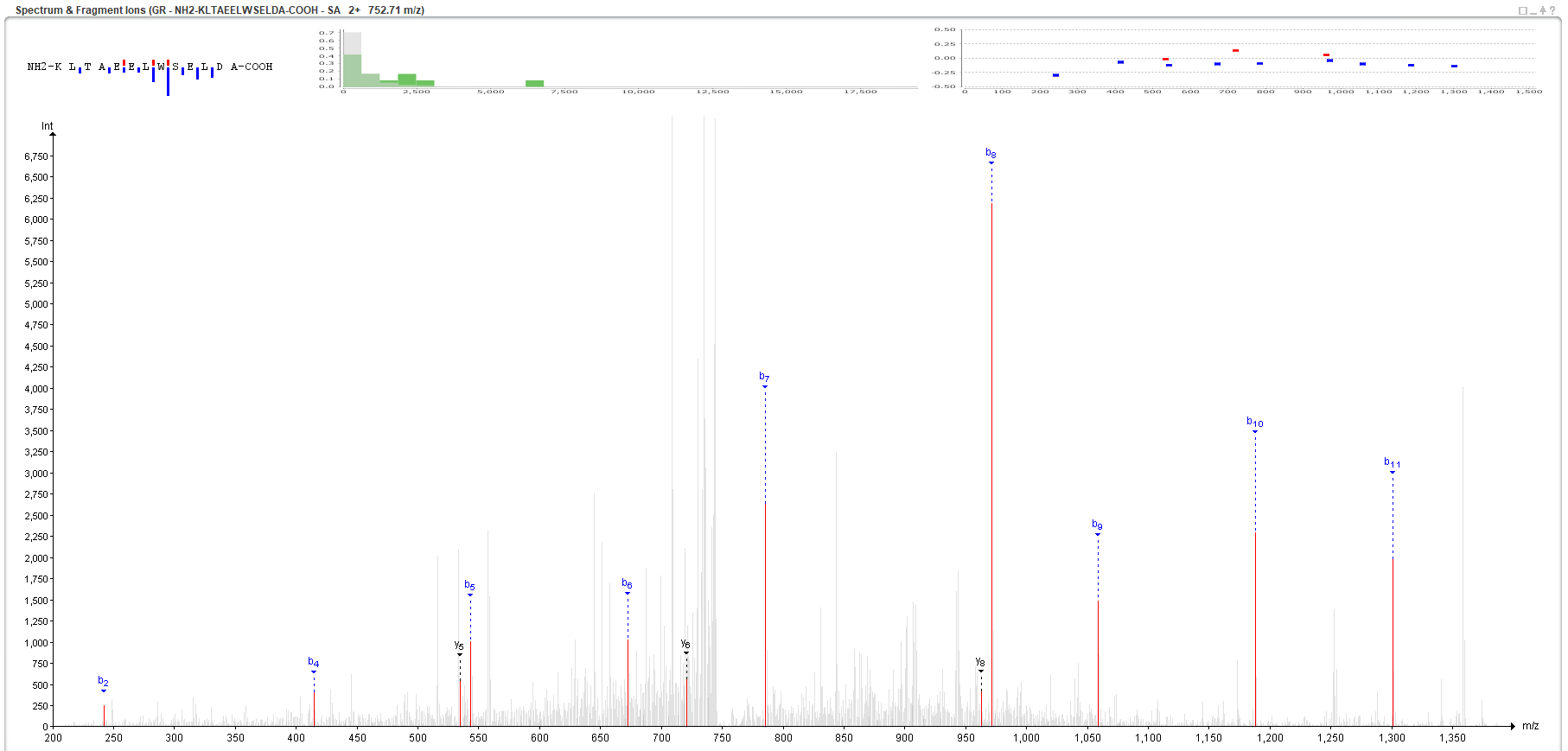


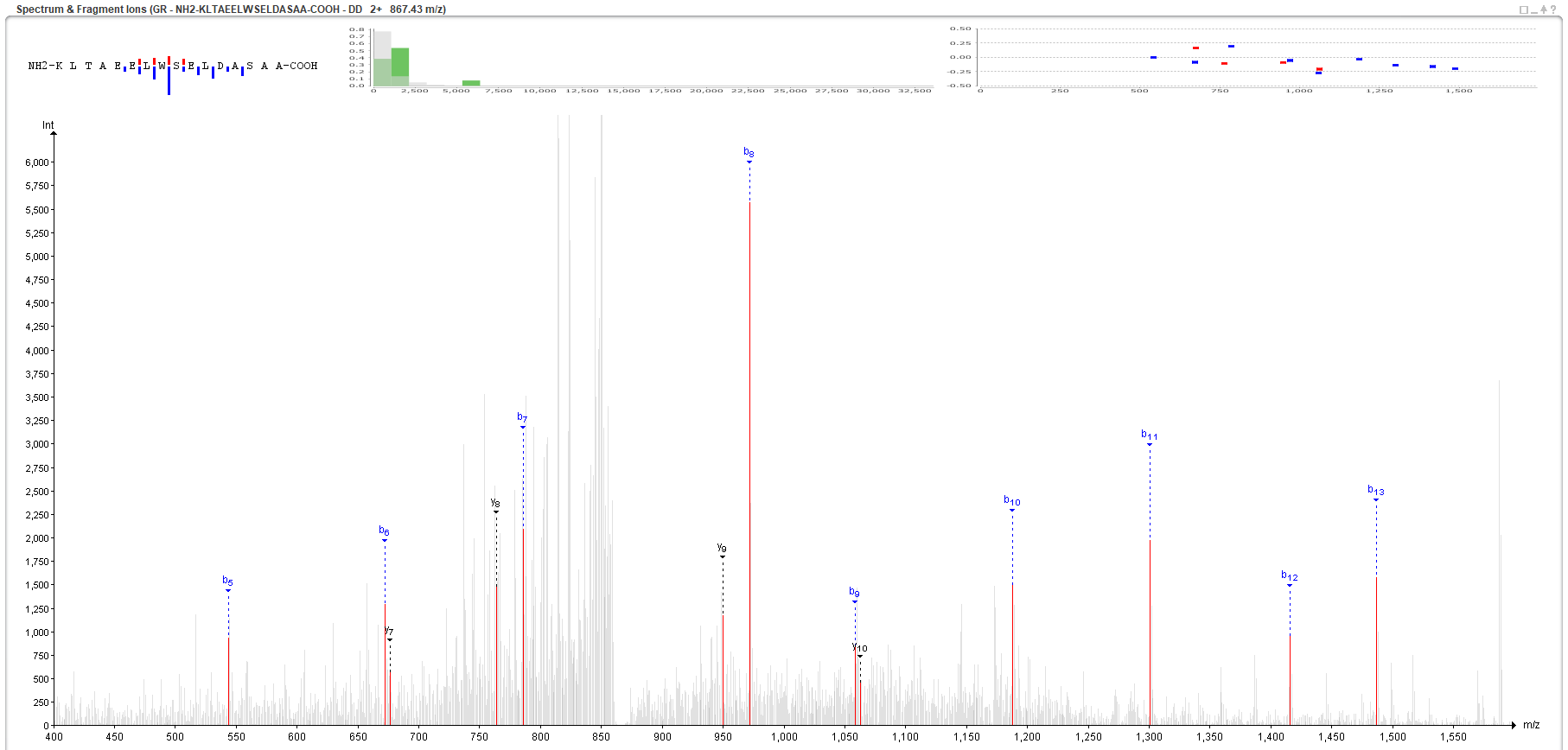


RAP2.12 expressed in wheat germ extract

**RCGGAIISDF IPPPRSRRVT SEFIWPDLKK** NLKGSKKSSK NR**SNFFDFDA EFEADFQGFK** DDSSIDCDDD FDVGDVFADV KPFVFTSTPK **PAVSAAAEGS VFGKKVTGLD GDAEK**SANRK RKNQYRGIRQ RPWGKWAAEI RDPREGAR**IW LGTFK**TAEEA ARAYDAAARR IRGSKAKVNF PEENMKANSQ KRSVKANLQK PVAKPNPNPS PALVQNSNIS FENMCFMEEK HQVSNNNNNQ FGMTNSVDAG CNGYQYFSSD QGSNSFDCSE FGWSDQAPIT PDISSAVINN NNSALFFEEA NPAKKLKSMD FETPYNNTEW DASLDFLNED AVTTQDNGAN PMDLWSIDEI HSMIGGVF


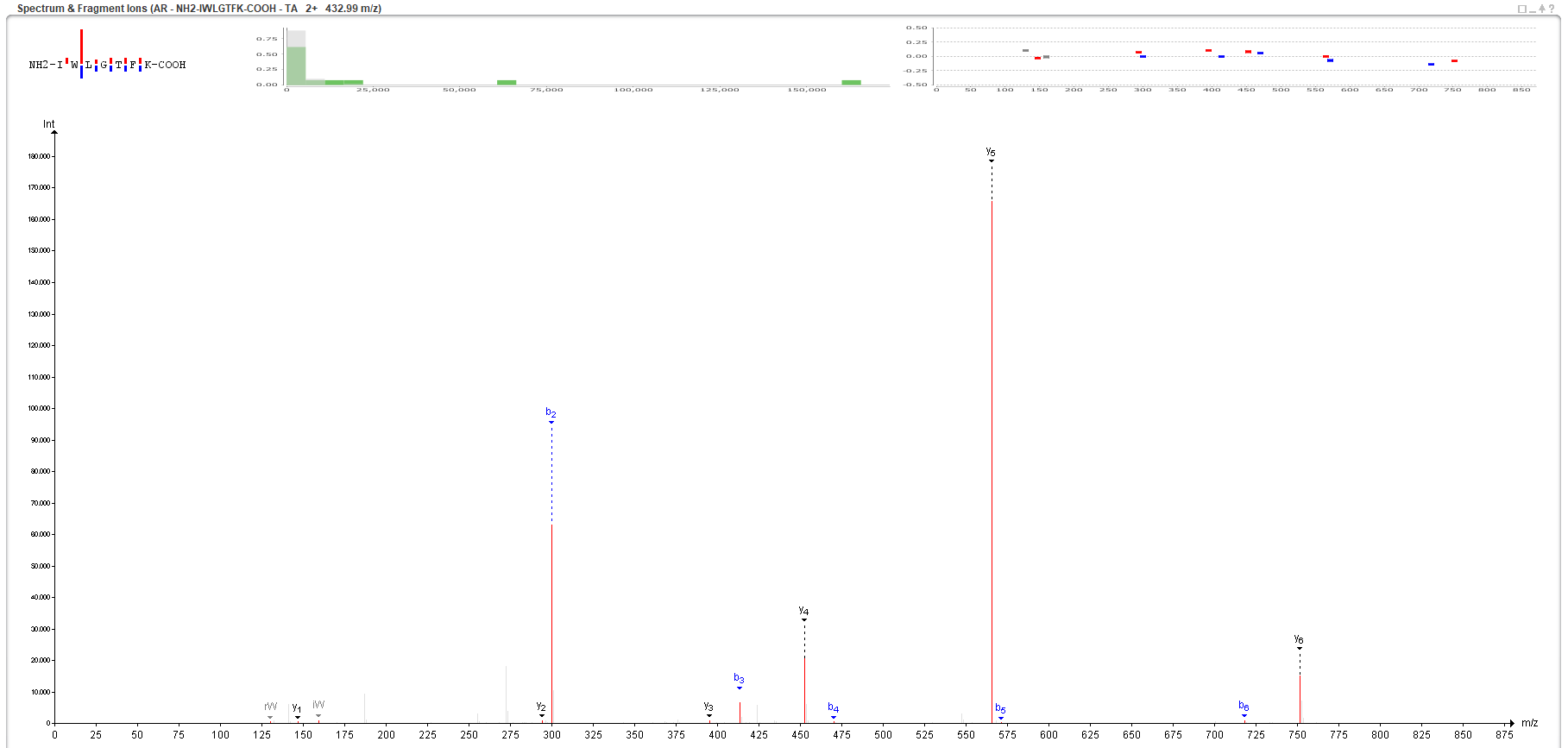


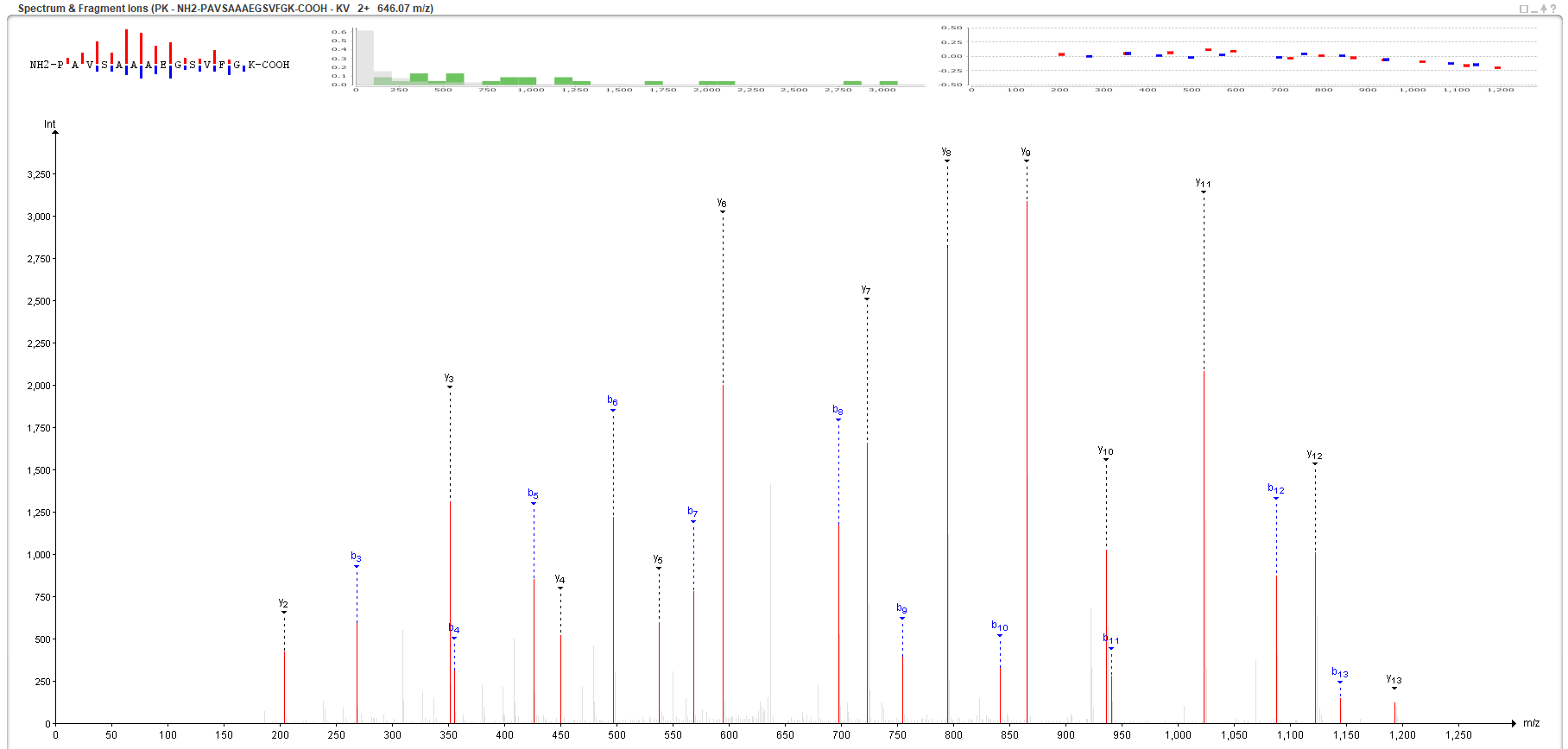


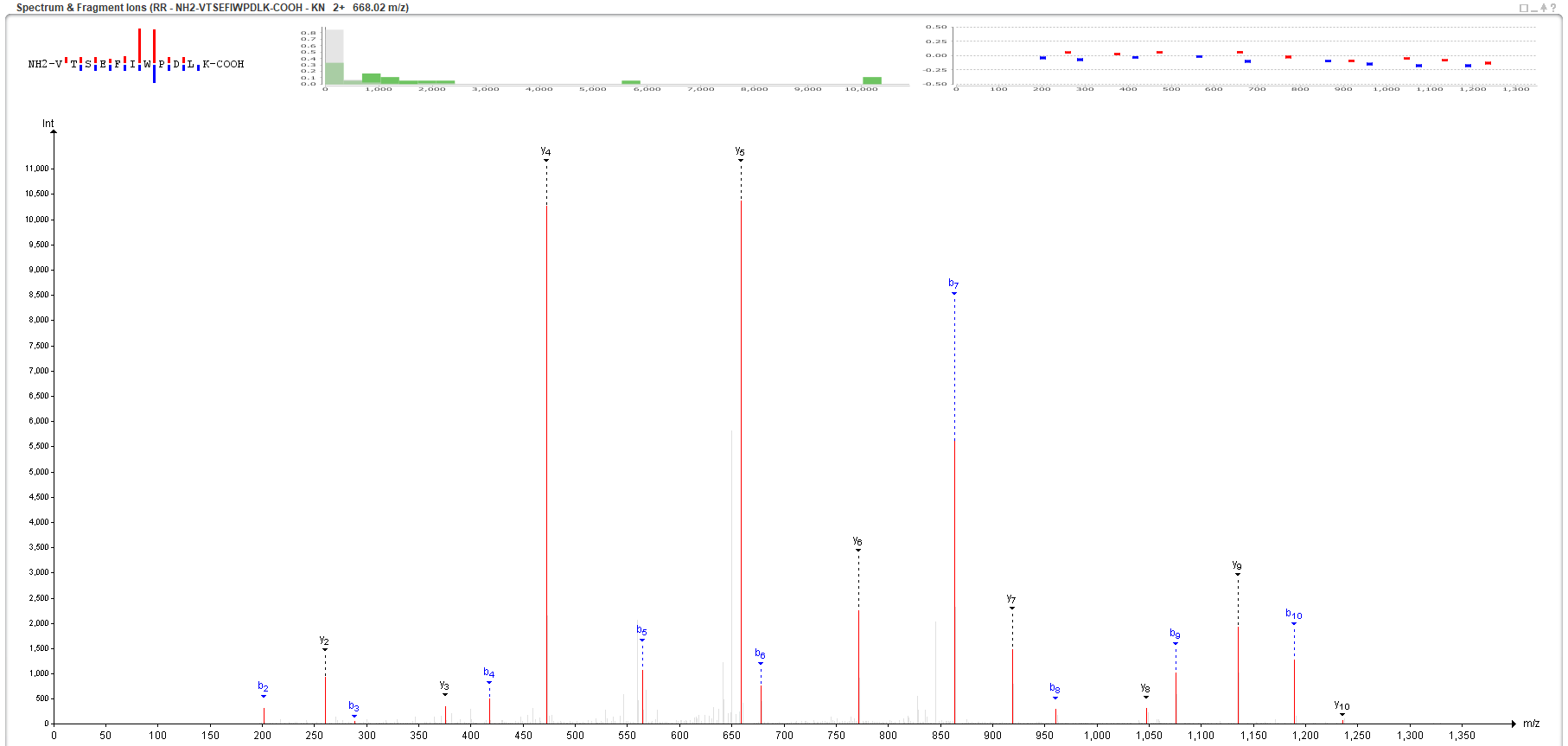


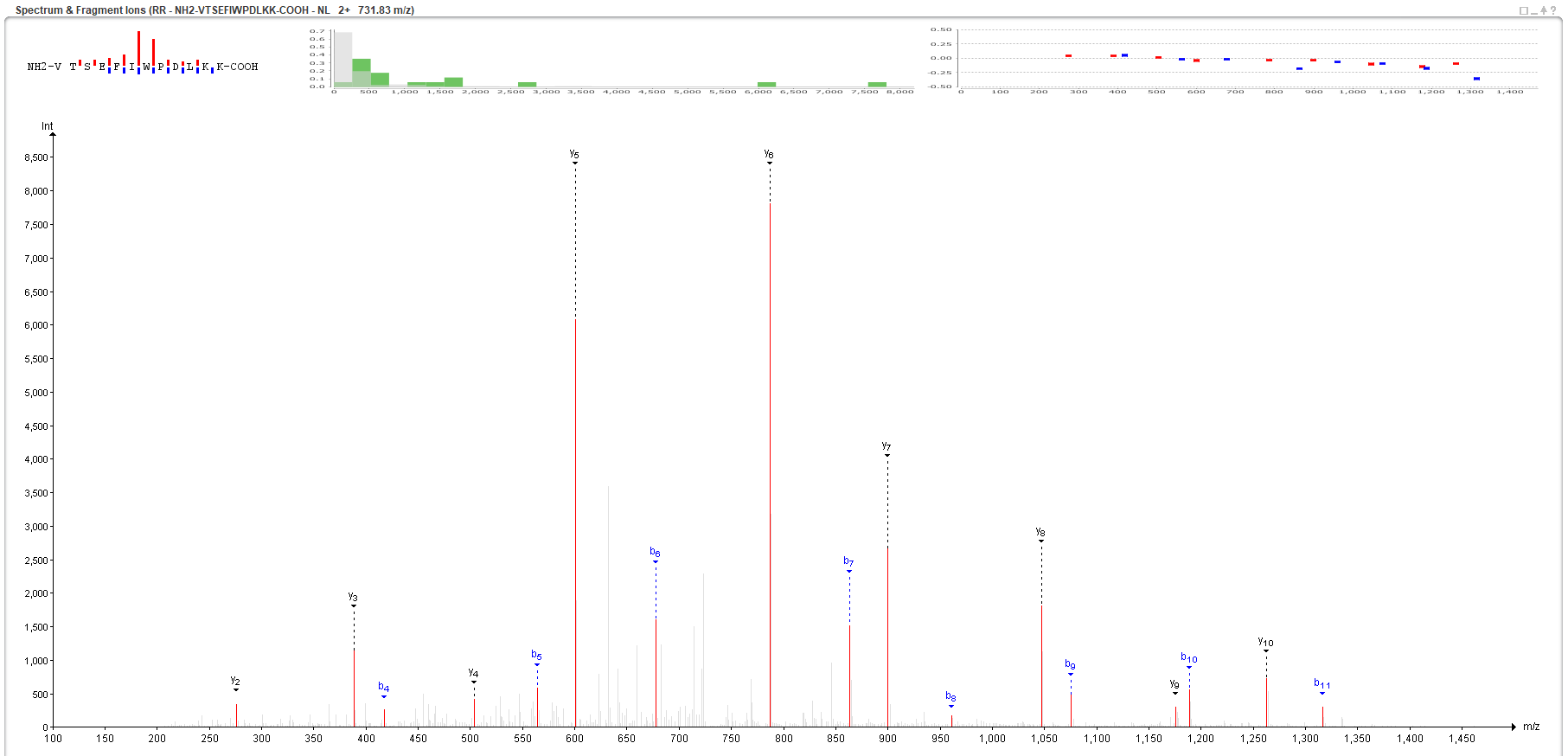


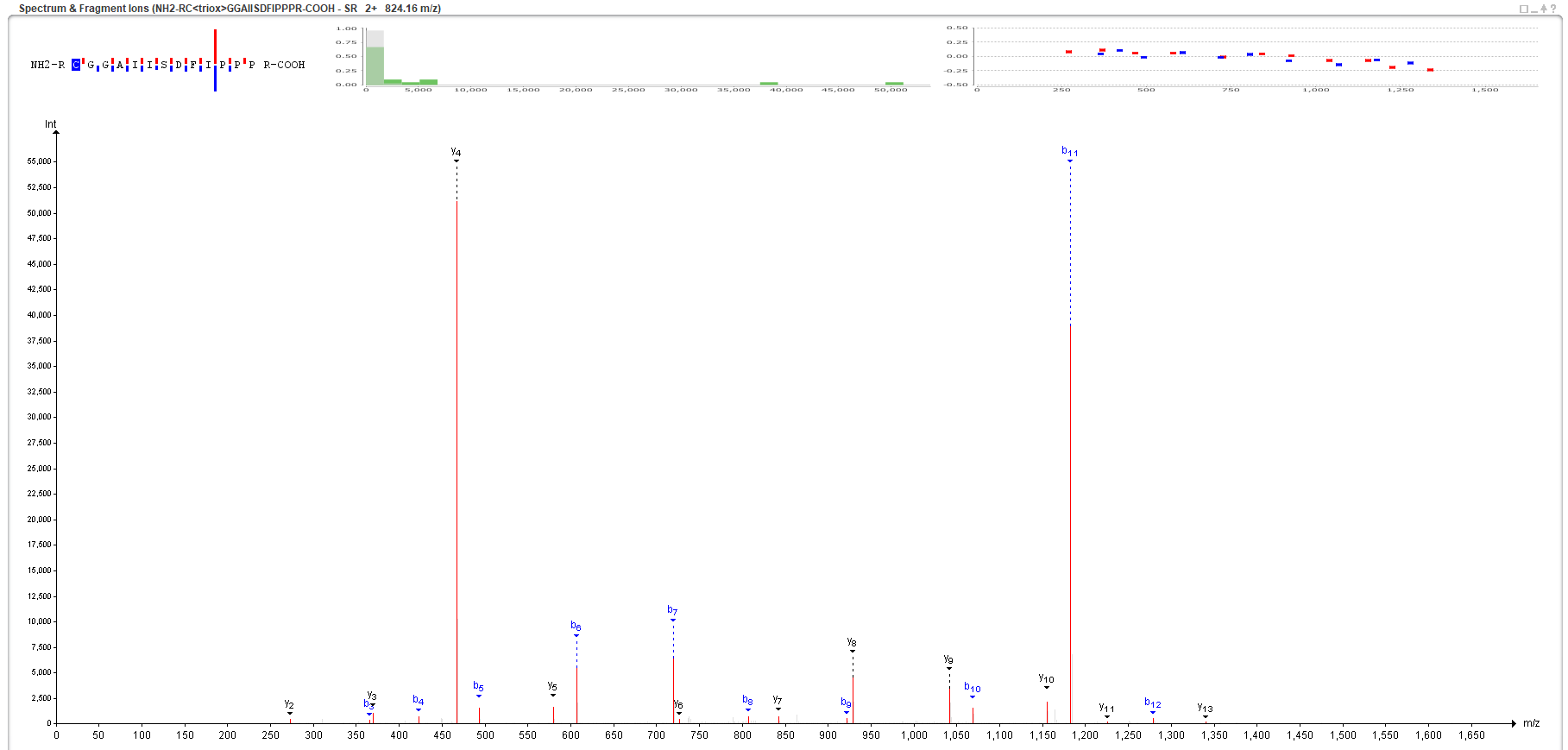


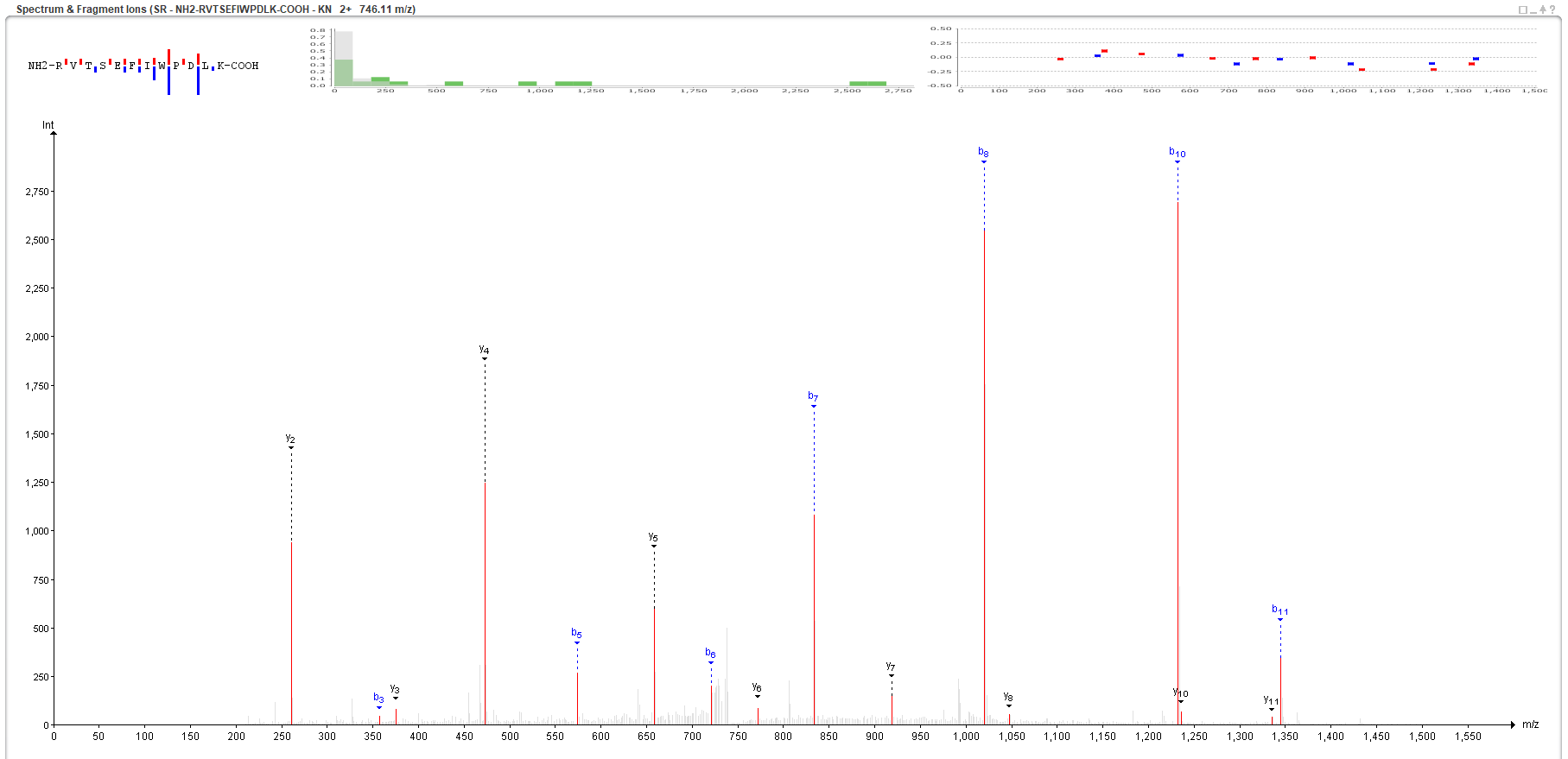


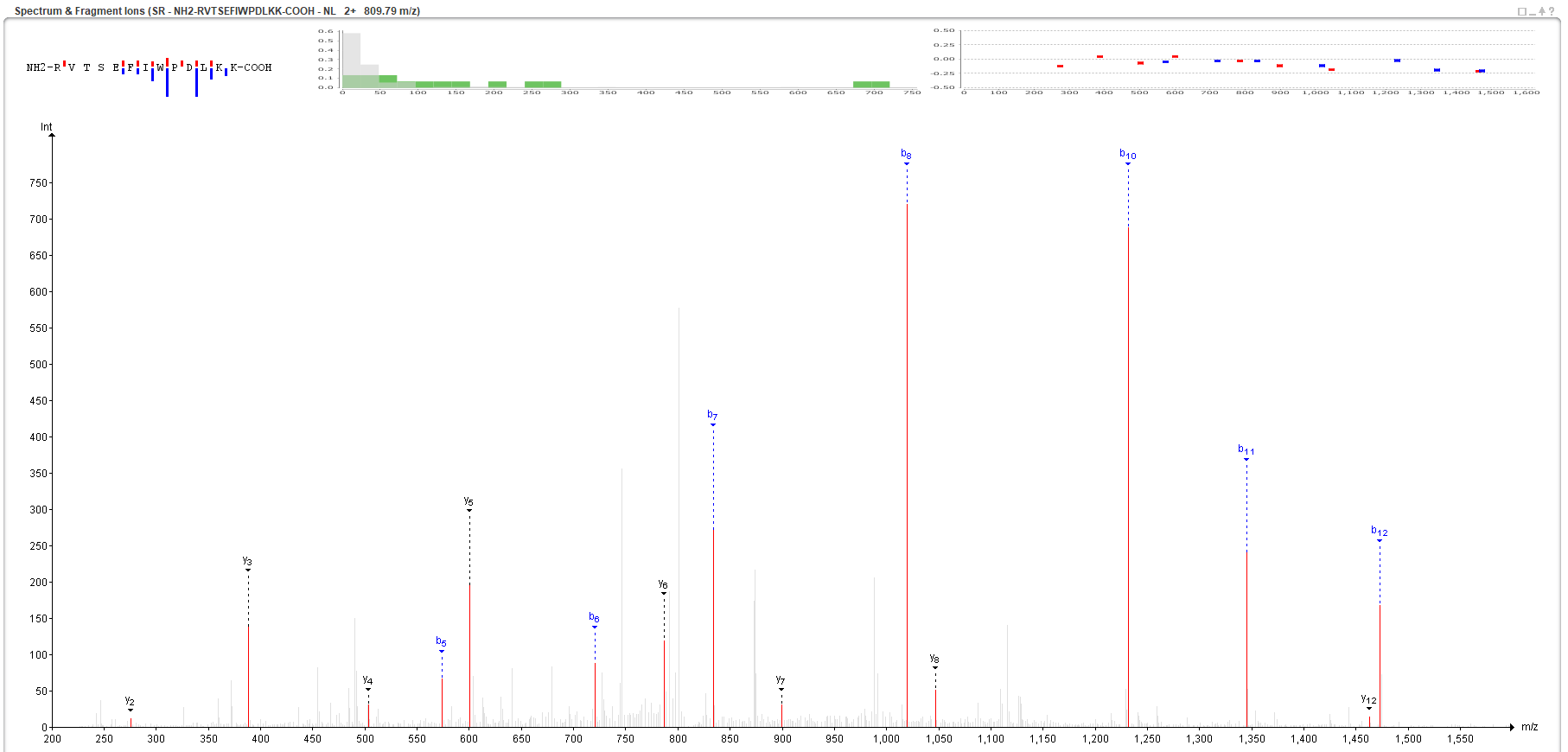


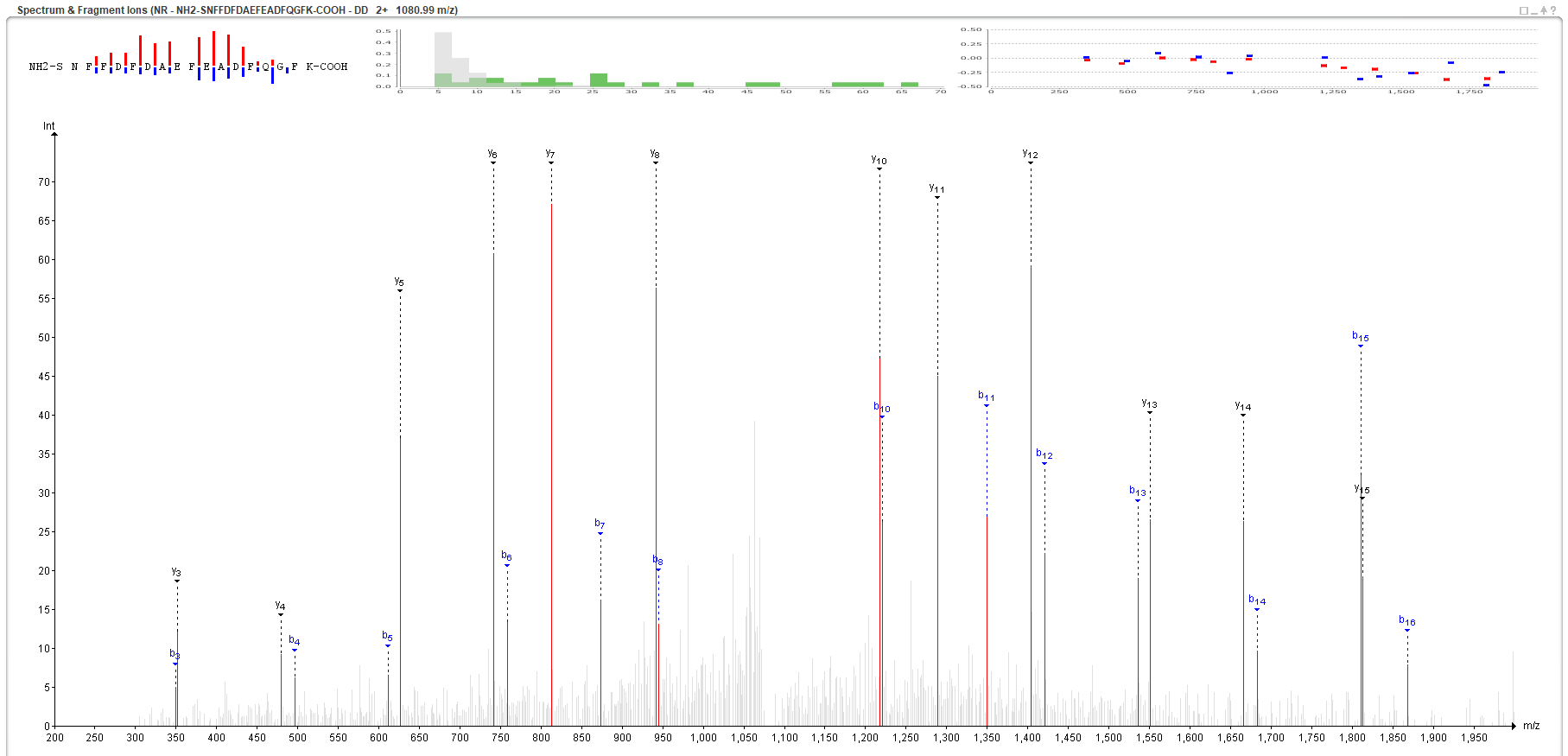


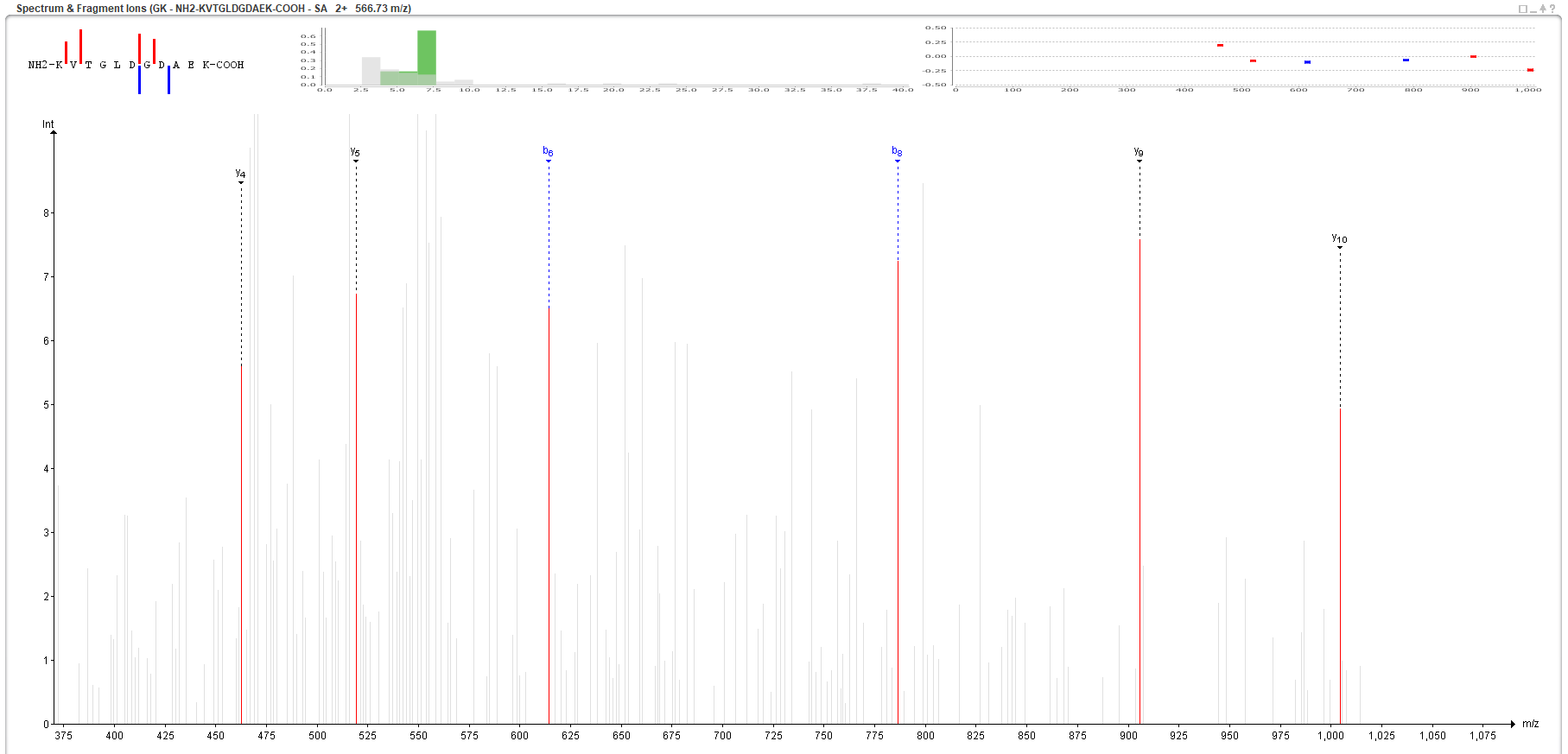


RAP2.3 expressed in E. coli

**CGGAIISDYA PLVTK**AKGRK LTAEELWSEL DASAADDFWG FYSTSKLHPT NQVNVKEEAV KKEQATEPGK RRKRKNVYRG IRKRPWGKWA AEIRDPRKGV R**VWLGTFNTA EEAAMAYDVA AK**QIRGDKAK LNFPDLHHPP PPNYT**PPPSS PR**STDQPPAK KVCVVSQSES ELSQPSFPVE CIGFGN**GDEF QNLSYGFEPD YDLKQQISSL ESFLELDGNT AEQ**PSQLDES VSEVDMWMLD DVIASYE


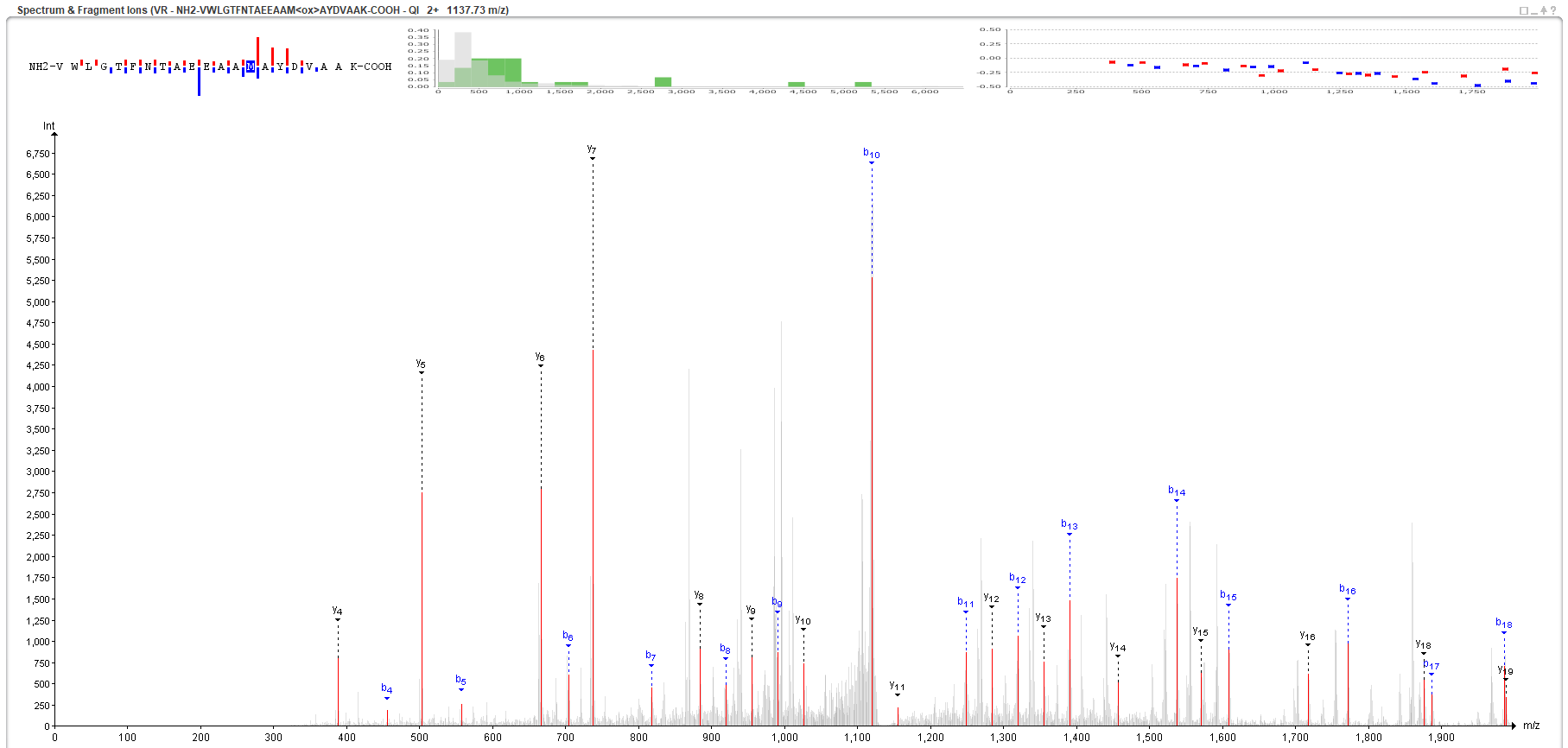


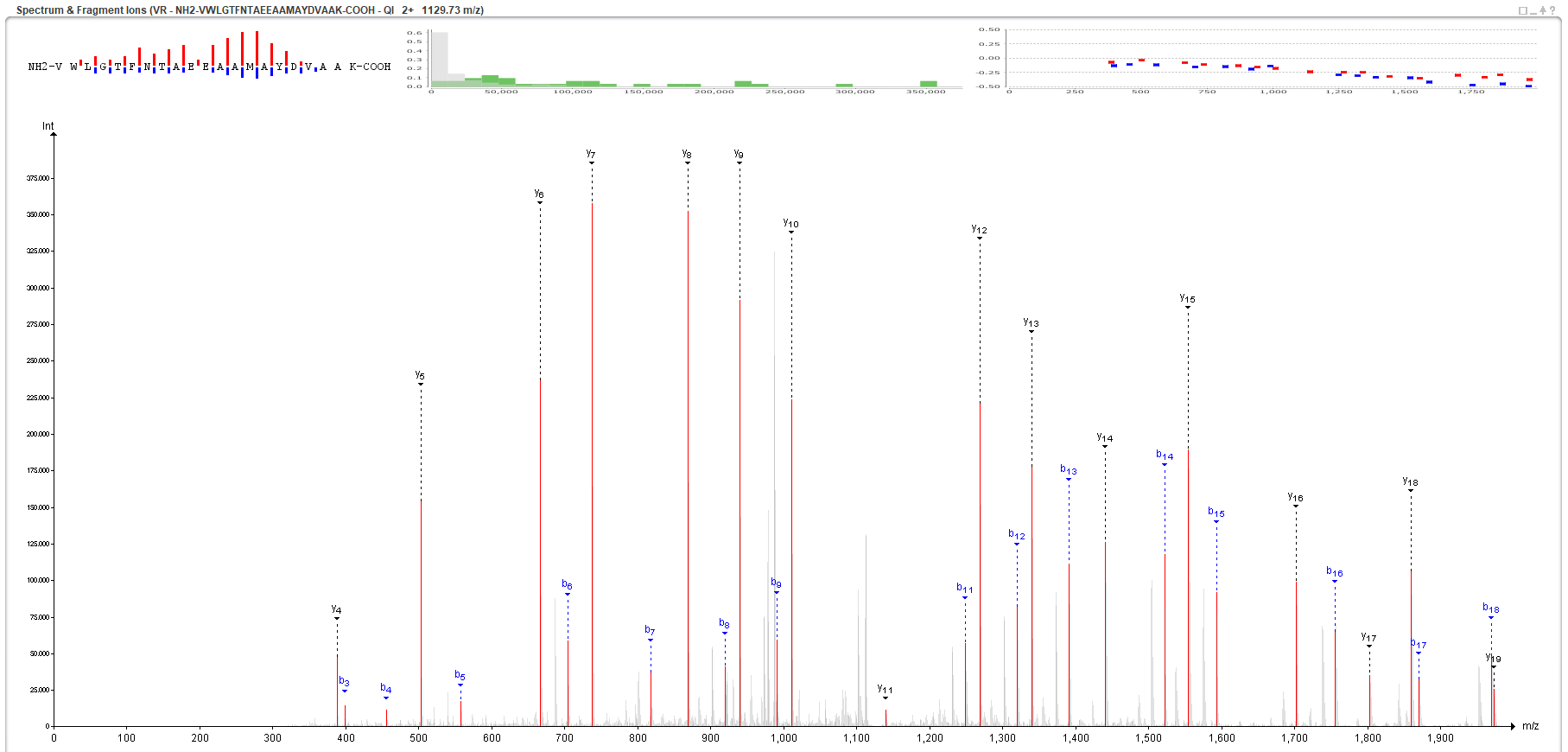


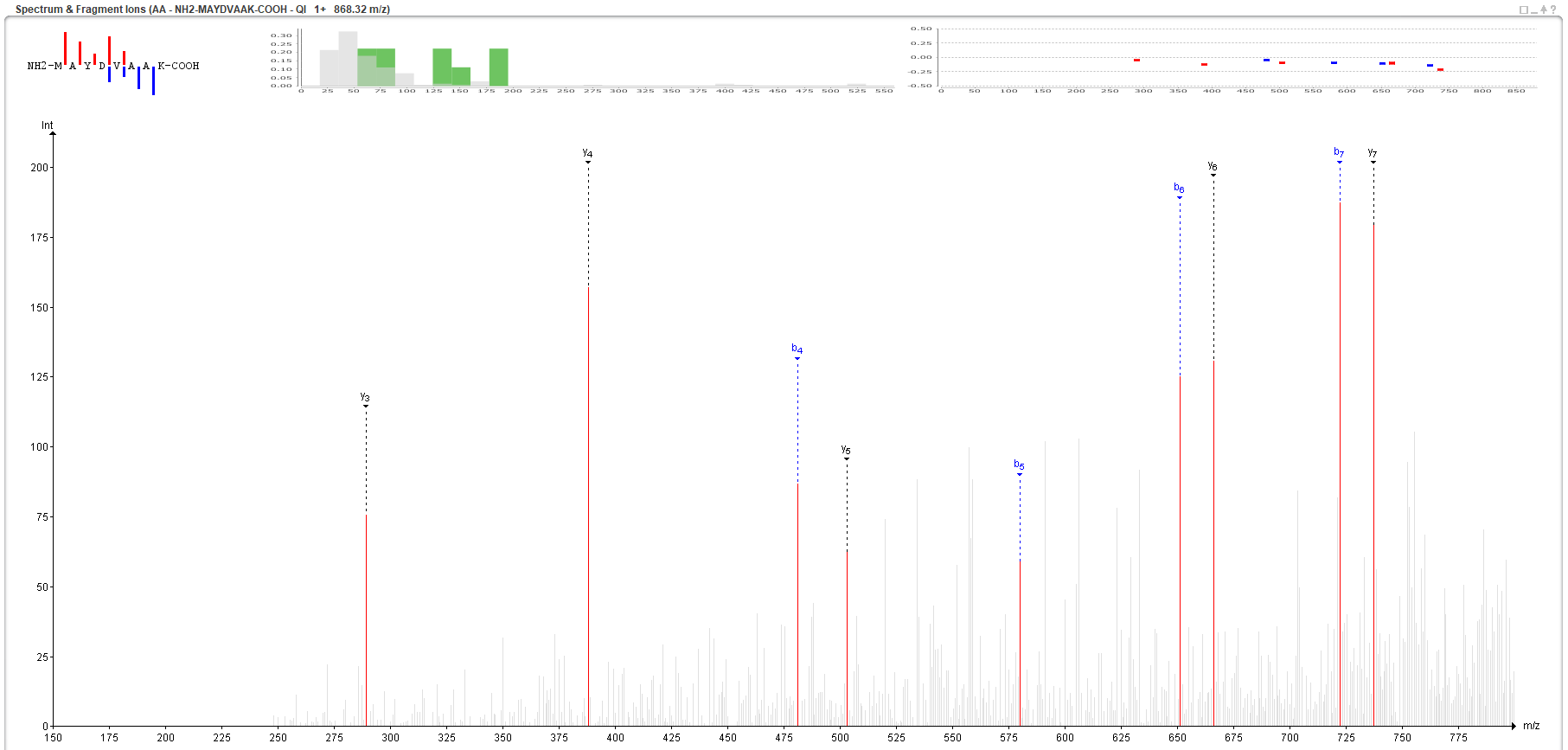


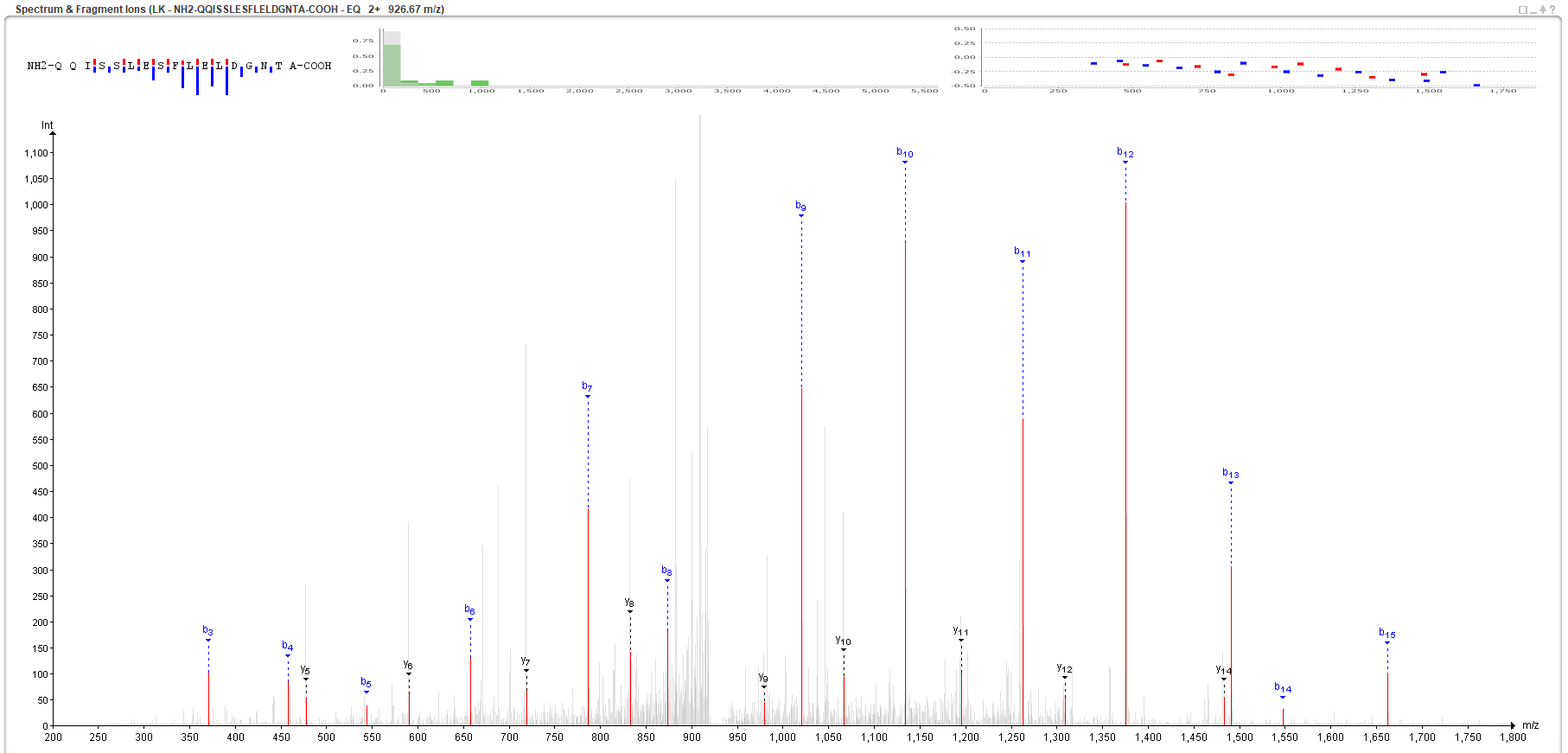


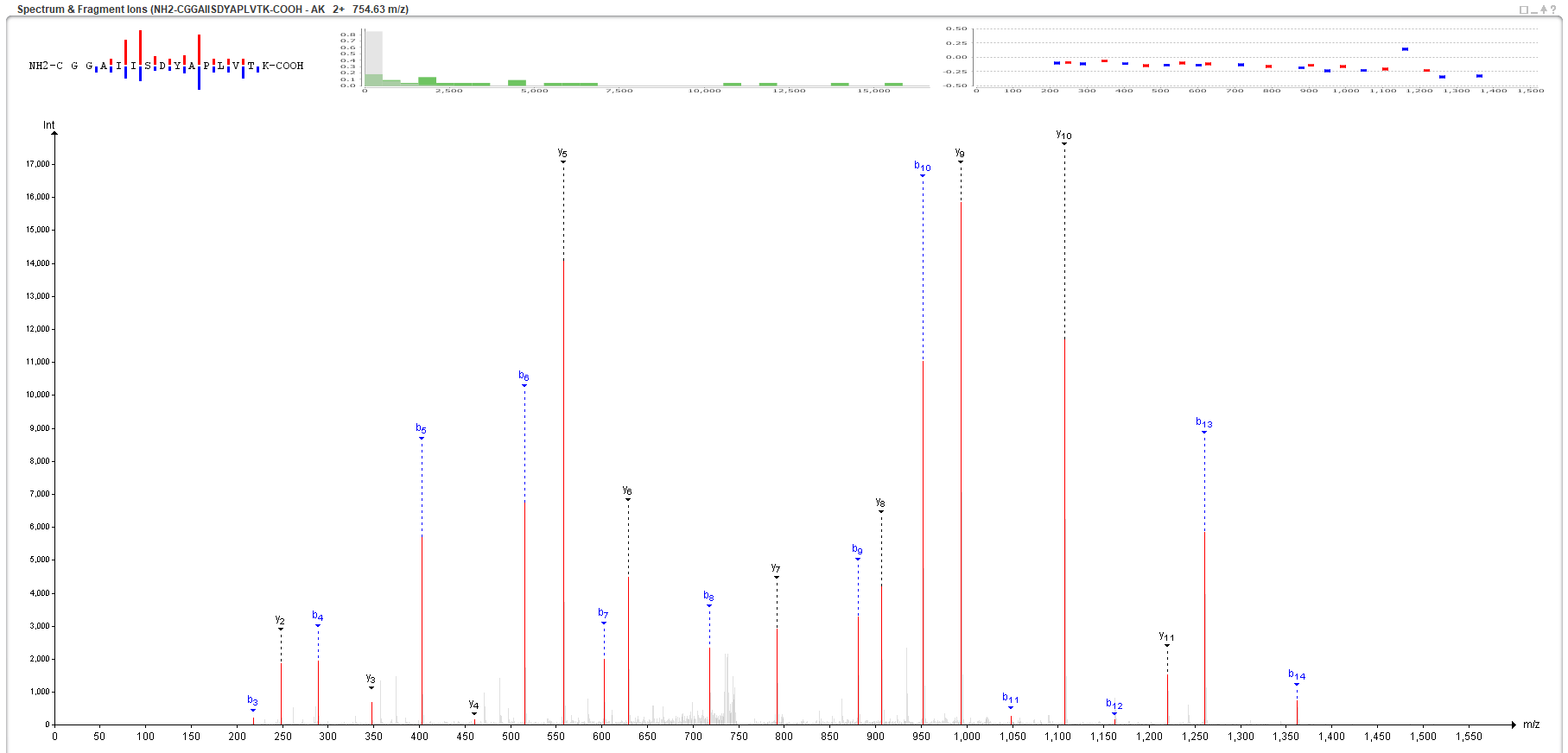


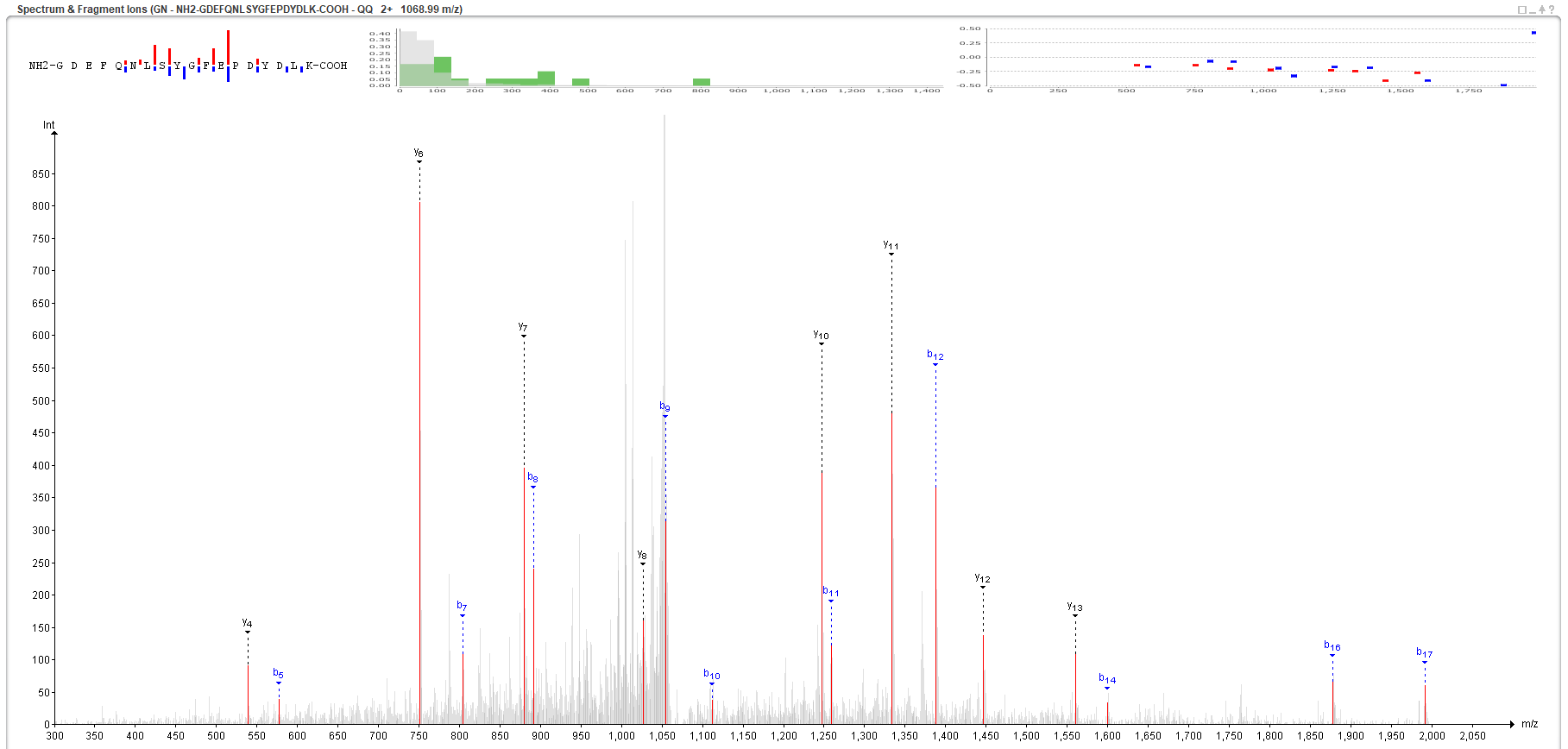


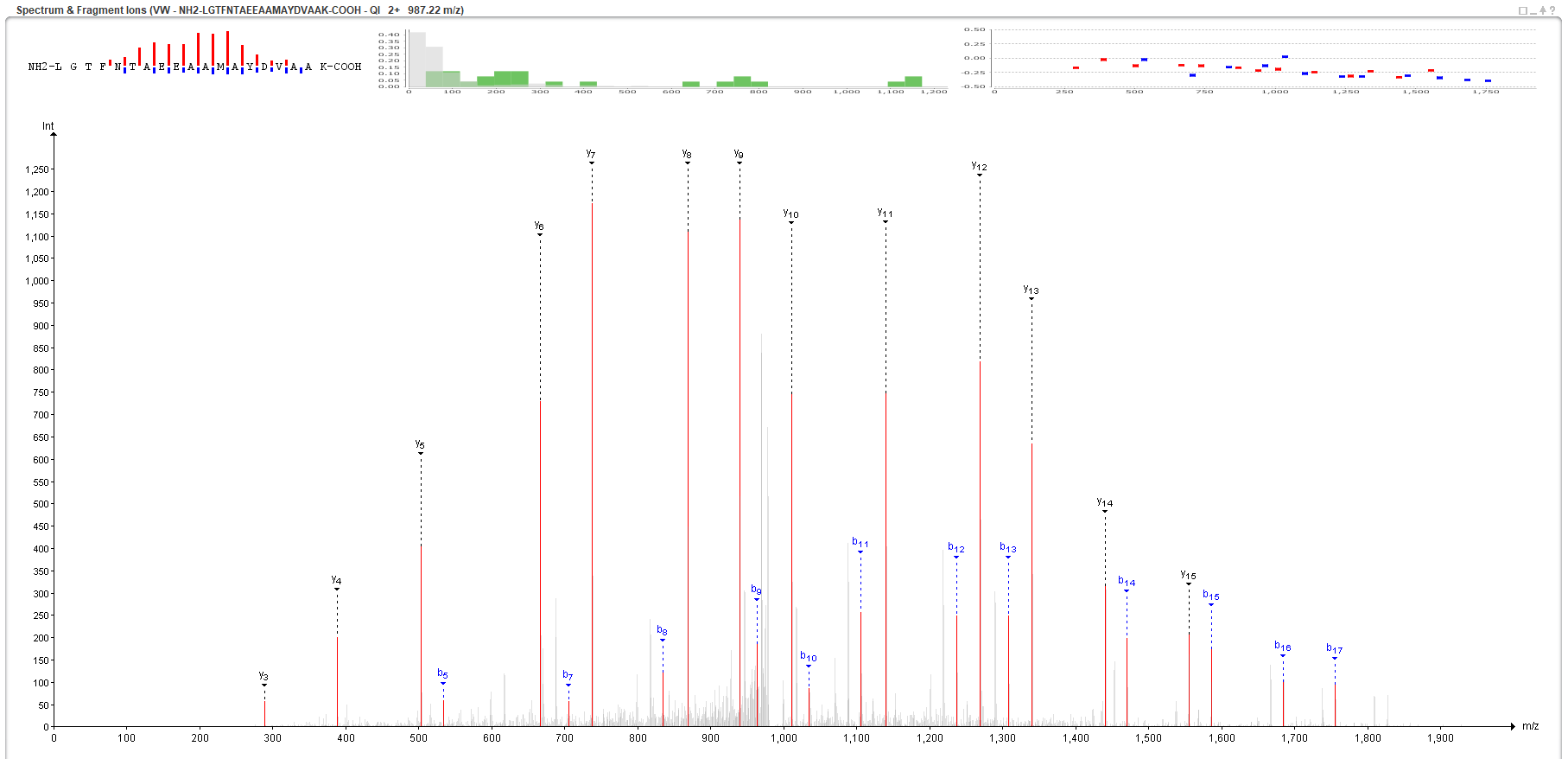


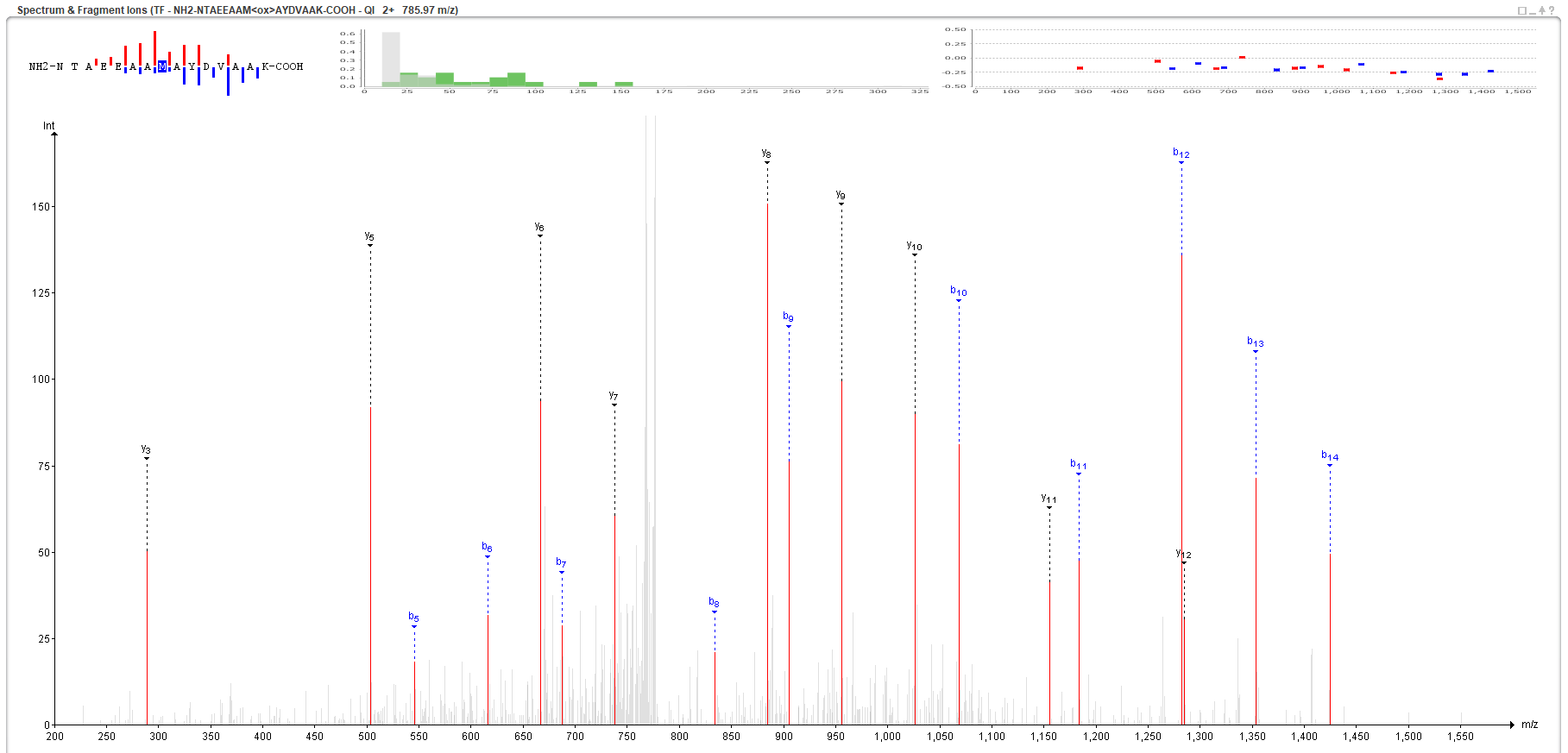


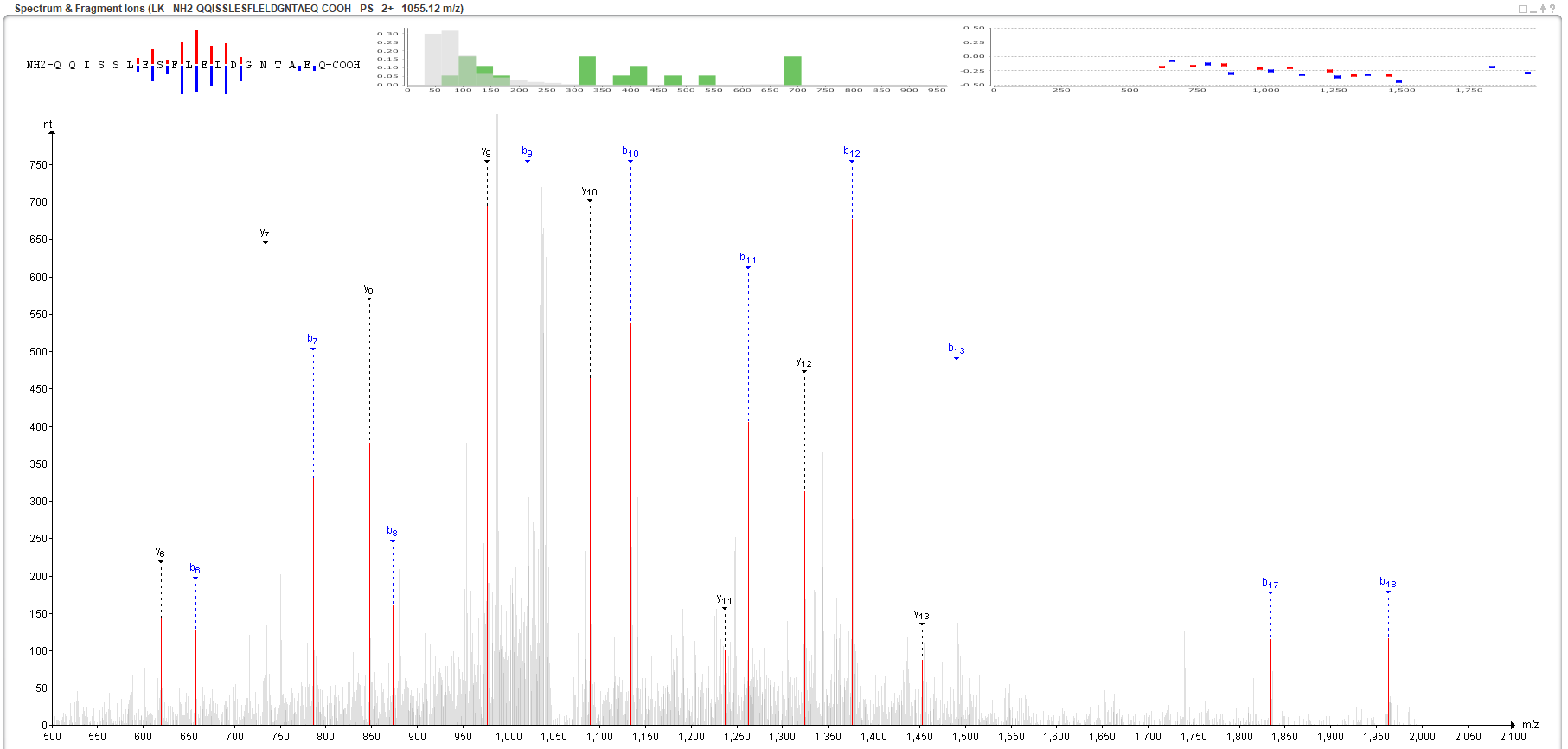


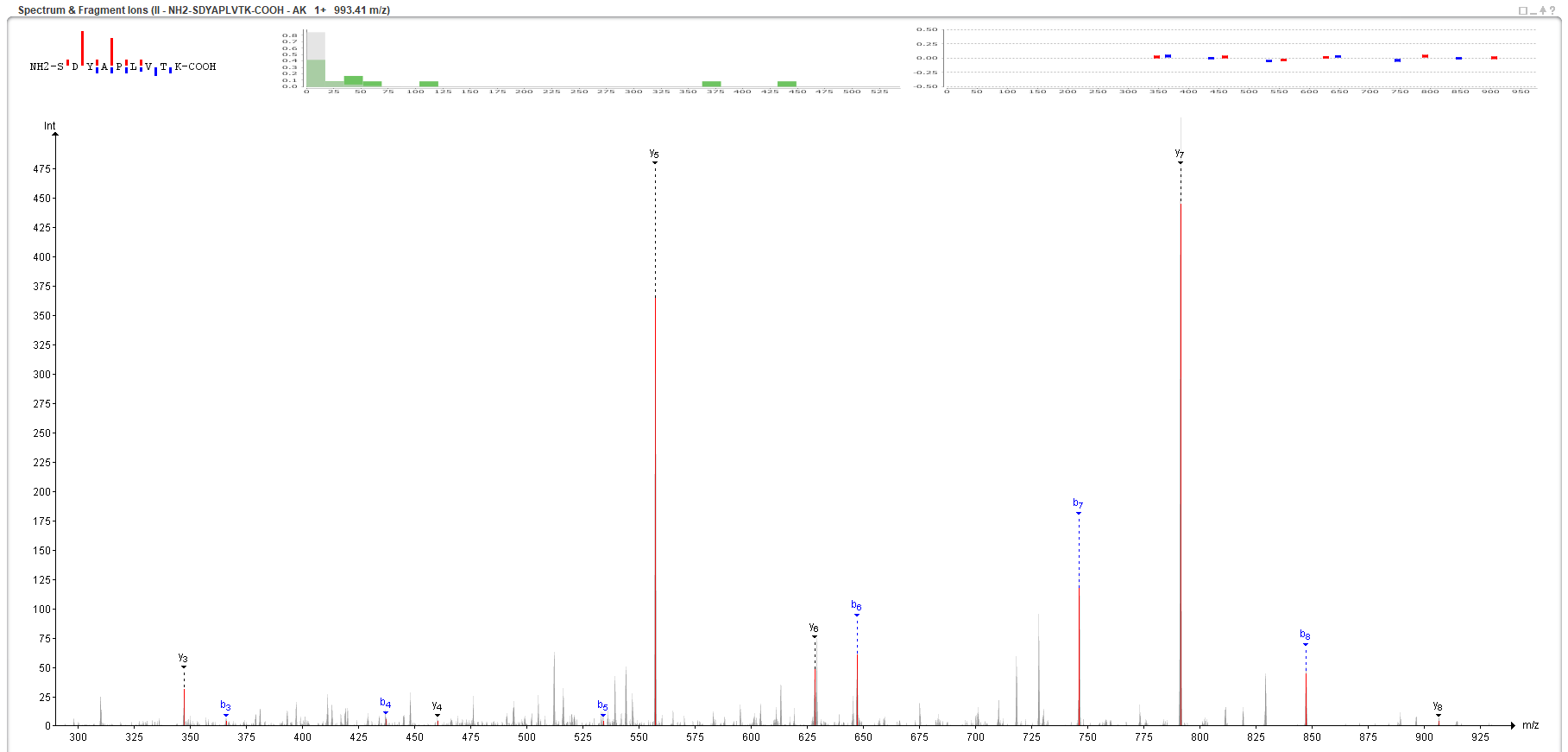


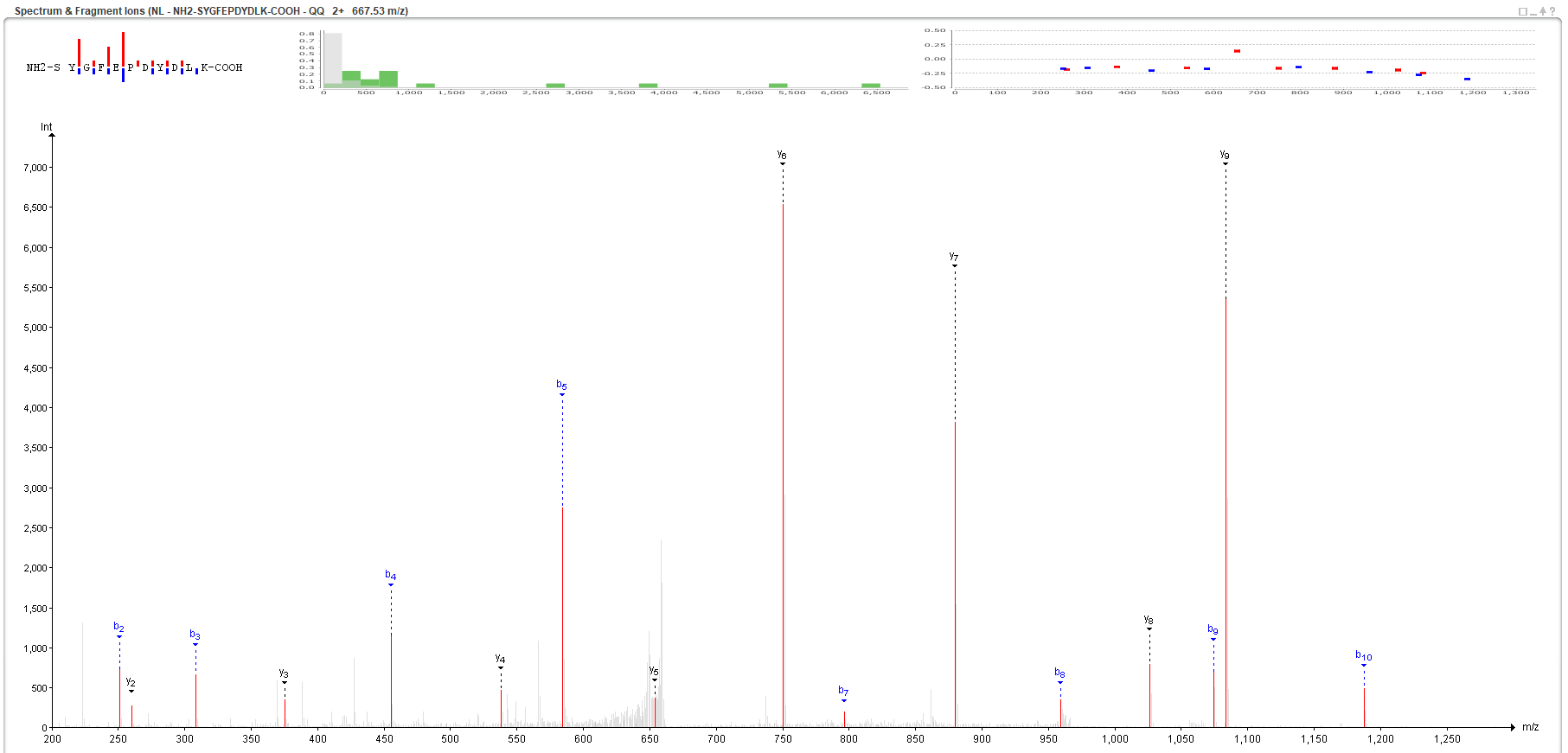


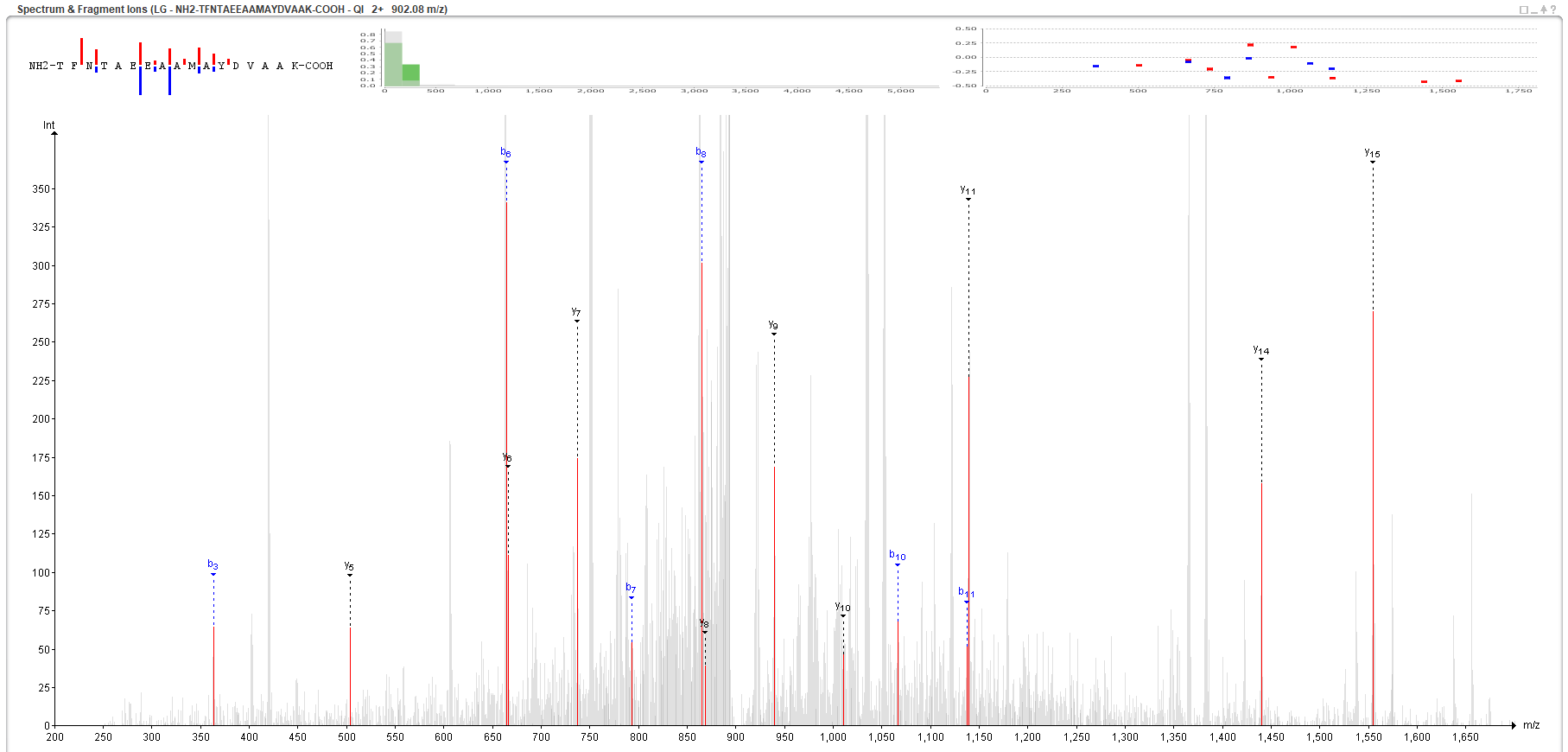


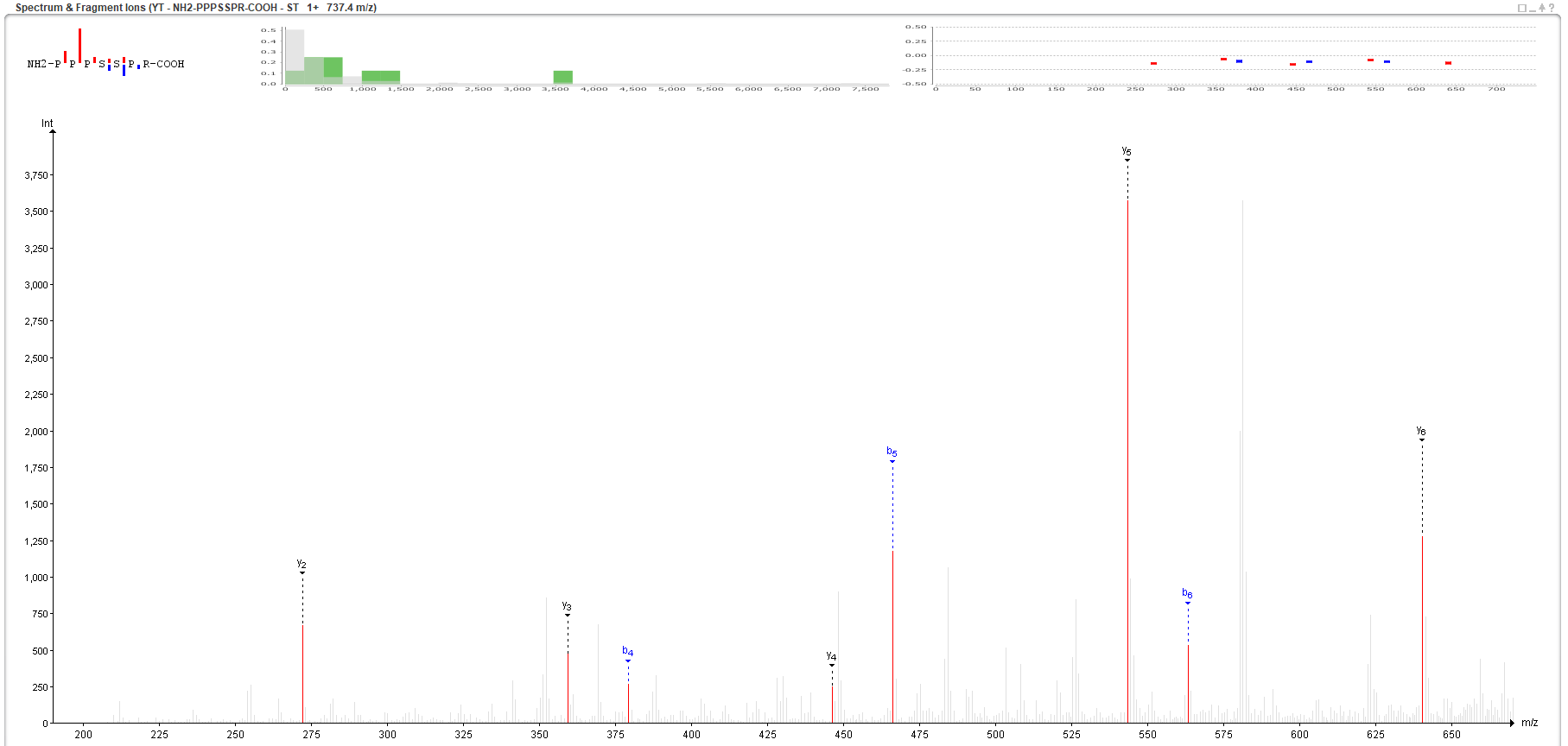


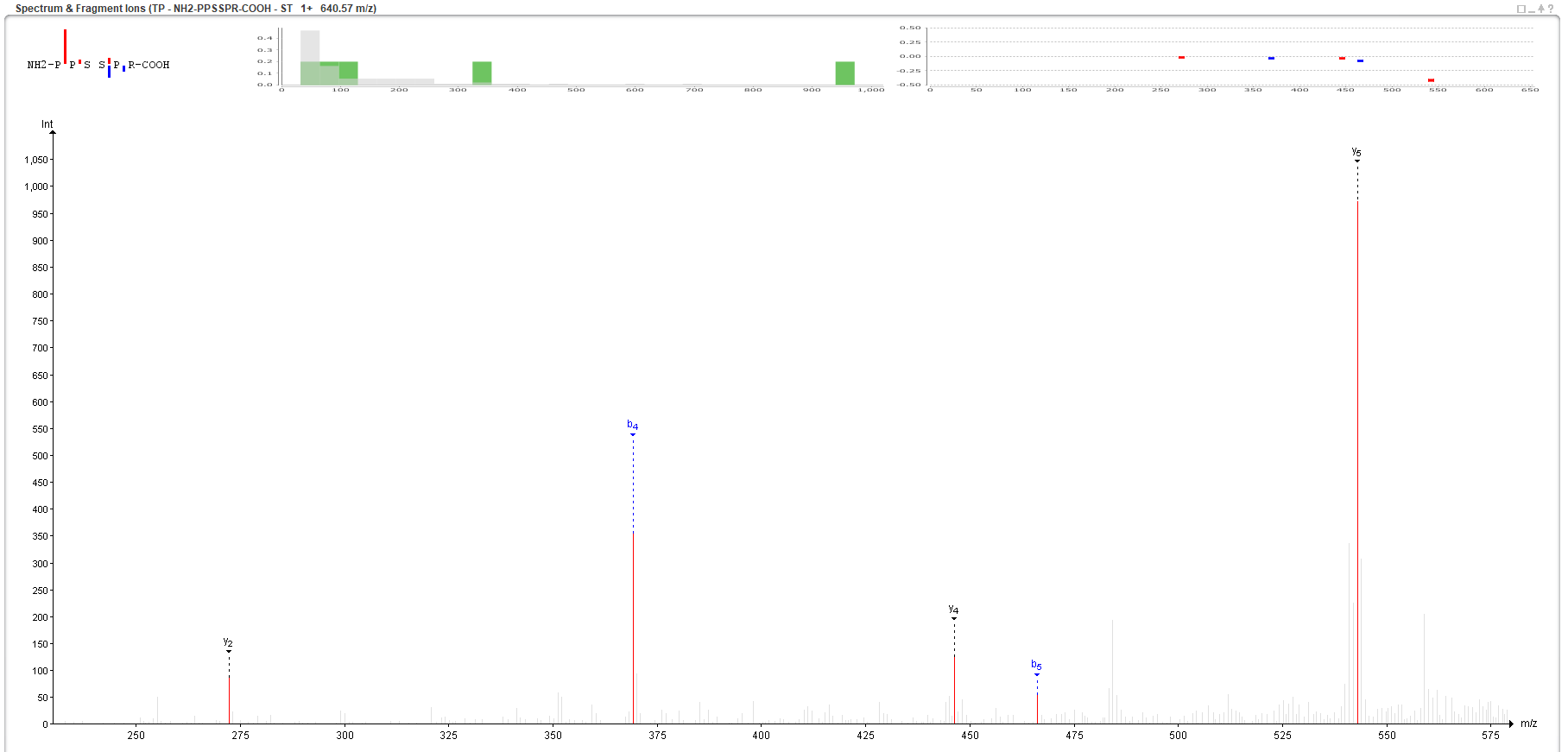

Supplement: Supplementary file 15 — Supplementary Data 12 [file 41467_2023_40366_MOESM15_ESM.docx]
