# Supplementary material for: ERFVII action and modulation through oxygen-sensing in Arabidopsis thaliana
Source: Nat Commun. 2023 Aug 3;14:4665. doi: 10.1038/s41467-023-40366-y (PMC10400637; doi:10.1038/s41467-023-40366-y)
Supplement: Supplementary file 19 — Reporting Summary [file 41467_2023_40366_MOESM19_ESM.pdf]

nature portfolio

Corresponding author(s): M J Holdsworth

Last updated by author(s): Mar 3, 2023

Reporting Summary

Nature Portfolio wishes to improve the reproducibility of the work that we publish. This form provides structure for consistency and transparency in reporting. For further information on Nature Portfolio policies, see our Editorial Policies and the Editorial Policy Checklist.

Statistics

For all statistical analyses, confirm that the following items are present in the figure legend, table legend, main text, or Methods section.

n/a	Confirmed	
☐	☒	The exact sample size (*n*) for each experimental group/condition, given as a discrete number and unit of measurement
☐	☒	A statement on whether measurements were taken from distinct samples or whether the same sample was measured repeatedly
☐	☒	The statistical test(s) used AND whether they are one- or two-sided<br>*Only common tests should be described solely by name; describe more complex techniques in the Methods section.*
☒	☐	A description of all covariates tested
☐	☒	A description of any assumptions or corrections, such as tests of normality and adjustment for multiple comparisons
☐	☒	A full description of the statistical parameters including central tendency (e.g. means) or other basic estimates (e.g. regression coefficient) AND variation (e.g. standard deviation) or associated estimates of uncertainty (e.g. confidence intervals)
☐	☒	For null hypothesis testing, the test statistic (e.g. *F*, *t*, *r*) with confidence intervals, effect sizes, degrees of freedom and *P* value noted<br>*Give P values as exact values whenever suitable.*
☒	☐	For Bayesian analysis, information on the choice of priors and Markov chain Monte Carlo settings
☒	☐	For hierarchical and complex designs, identification of the appropriate level for tests and full reporting of outcomes
☒	☐	Estimates of effect sizes (e.g. Cohen's *d*, Pearson's *r*), indicating how they were calculated

Our web collection on statistics for biologists contains articles on many of the points above.

Software and code

Policy information about availability of computer code

Data collection	*Provide a description of all commercial, open source and custom code used to collect the data in this study, specifying the version used OR state that no software was used.*
Data analysis	Prism Graphpad (v.9), Microsoft Excel (v16), VGStudioMAX (version 2.2.0; Volume Graphics GmbH), DatosRec software (Waygate Technologies), skewer v0.2.2, Hisat2 v2.1.0, featureCounts v1.6.0, DESeq2 v1.24.0, GOSeq v1.4.0, G:Profiler, MEME Suite 5.4.1 FIMO, Xcalibur, http://db.systemsbiology.net:8080/proteomicsToolkit/FragIonServlet.html

For manuscripts utilizing custom algorithms or software that are central to the research but not yet described in published literature, software must be made available to editors and reviewers. We strongly encourage code deposition in a community repository (e.g. GitHub). See the Nature Portfolio guidelines for submitting code & software for further information.

Data

Policy information about availability of data

All manuscripts must include a data availability statement. This statement should provide the following information, where applicable:

- Accession codes, unique identifiers, or web links for publicly available datasets
- A description of any restrictions on data availability
- For clinical datasets or third party data, please ensure that the statement adheres to our policy

The RNAseq data generated in this study have been deposited in the GEO database database under accession code GSE224694. The mass spectrometry proteomics data used in this study are available in the ProteomeXchange Consortium via the PRIDE 74 partner repository with the dataset identifier PXD039288. Source data

are provided with this paper.

Human research participants

Policy information about studies involving human research participants and Sex and Gender in Research.

Reporting on sex and gender	NA
Population characteristics	NA
Recruitment	NA
Ethics oversight	NA

Note that full information on the approval of the study protocol must also be provided in the manuscript.

Field-specific reporting

Please select the one below that is the best fit for your research. If you are not sure, read the appropriate sections before making your selection.

[x] Life sciences [ ] Behavioural & social sciences [ ] Ecological, evolutionary & environmental sciences

For a reference copy of the document with all sections, see nature.com/documents/nr-reporting-summary-flat.pdf

Life sciences study design

All studies must disclose on these points even when the disclosure is negative.

Sample size	We used at least three independent biological samples size following standard practice in the field.
Data exclusions	no data were excluded from the analyses
Replication	Data were collected from at least three independent replications for each experiment, and replication was successful and showed comparable trends. All experiments wer also replicated at least twice with similar observations.
Randomization	For phenotyping and gene expression analyses of different genetic materials samples were assayed and processed randomly
Blinding	Investigators were not blinded because this was not deemed necessary for our study.

Reporting for specific materials, systems and methods

We require information from authors about some types of materials, experimental systems and methods used in many studies. Here, indicate whether each material, system or method listed is relevant to your study. If you are not sure if a list item applies to your research, read the appropriate section before selecting a response.

Materials & experimental systems

n/a	Involved in the study	
[ ]	[x]	Antibodies
[x]	[ ]	Eukaryotic cell lines
[x]	[ ]	Palaeontology and archaeology
[x]	[ ]	Animals and other organisms
[x]	[ ]	Clinical data
[x]	[ ]	Dual use research of concern

Methods

n/a	Involved in the study	
[x]	[ ]	ChIP-seq
[x]	[ ]	Flow cytometry
[x]	[ ]	MRI-based neuroimaging

Antibodies

Antibodies used	For Westerns: Primary antibodies used were anti-HA (Sigma, H3663-200UL; 1:1000 dilution), anti-FLAG Sigma, F1804-200UG, 1:2000 dilution) and secondary antibody Goat anti-Mouse IgG1, HRP from Thermo Fisher Scientific, PA1 74421, (1:10000 dilution). For immundetection: Anti-HA primary antibody (Roche, Lewes, UK) and Alexa-Fluor-488-coupled anti rat secondary antibody (1:200 dilution) (Molecular Probes, Carlsbad, CA) were used for detection.

Validation	Validation of the antibodies has been reported by the producers and in many refereed scientific pubications
