# Supplementary material for: ERFVII action and modulation through oxygen-sensing in Arabidopsis thaliana
Source: Nat Commun. 2023 Aug 3;14:4665. doi: 10.1038/s41467-023-40366-y (PMC10400637; doi:10.1038/s41467-023-40366-y)
Supplement: Supplementary file 20 — Source Data [file 41467_2023_40366_MOESM20_ESM.zip › Uncropped Western blot images.pdf]

Uncropped Western blot images:

Red boxes indicate regions shown in figures

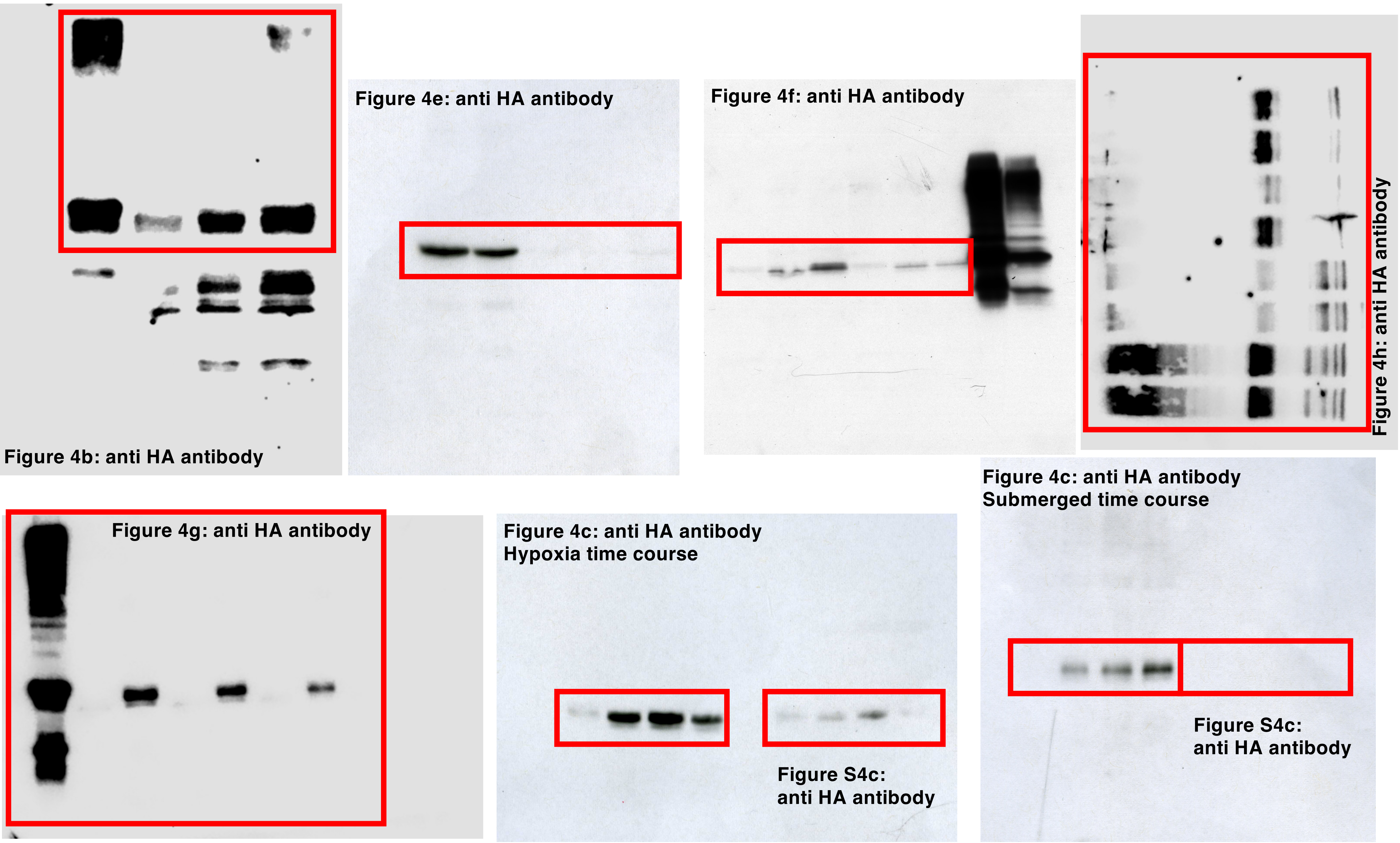

Uncropped Western blot images:

Red boxes indicate regions shown in figures

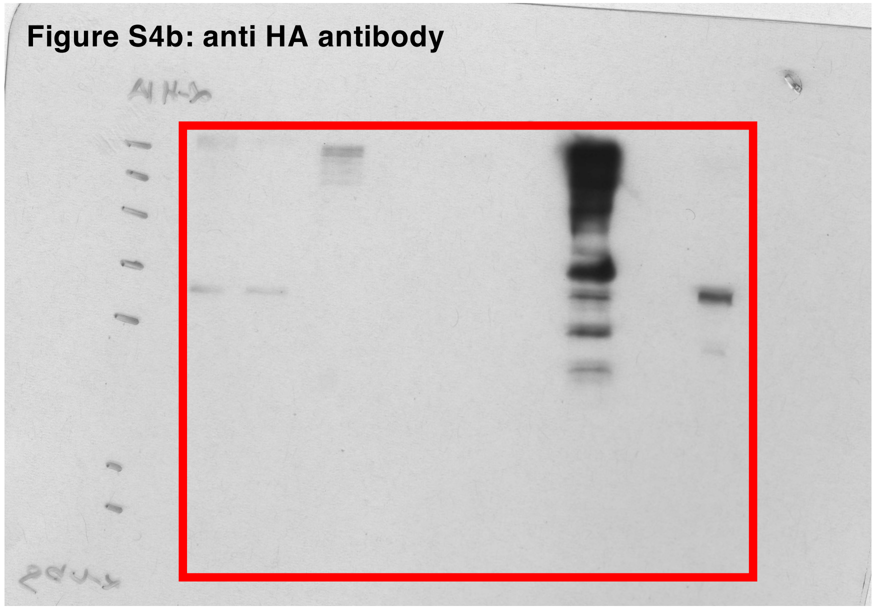

Figure S4b: anti HA antibody

Figure S4c: anti Ubiquitin antibody

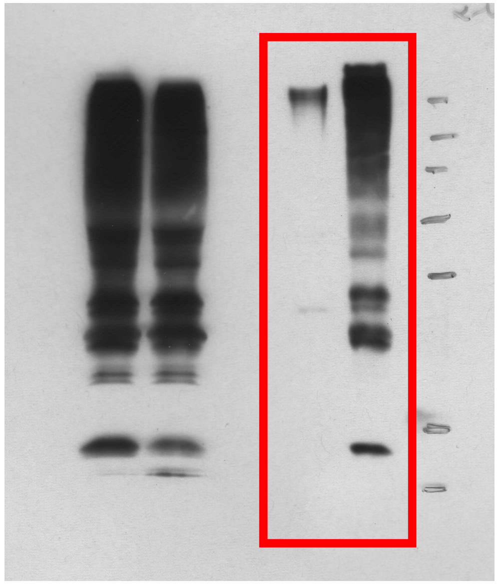

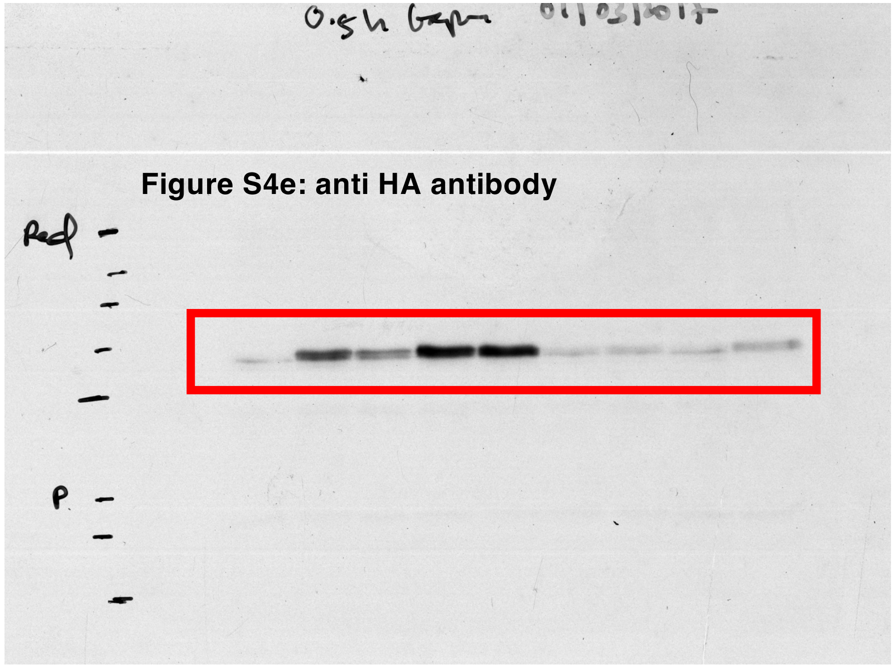

Figure S4e: anti HA antibody
